# Green pharmacy at the tips of your toes: medicinal plants used by Setos and Russians of Pechorsky District, Pskov Oblast (NW Russia)

**DOI:** 10.1186/s13002-022-00540-w

**Published:** 2022-06-17

**Authors:** Olga Belichenko, Valeria Kolosova, Raivo Kalle, Renata Sõukand

**Affiliations:** 1 Department of Environmental Sciences, Informatics and Statistics, Ca’ Foscari University of Venice, Via Torino 155, Mestre, 30172 Venice, Italy; 2 UMR 208 PALOC, Muséum National d’Histoire Naturelle, 47 rue Cuvier, 75005 Paris, France; 3 University of Gastronomic Sciences, Piazza Vittorio Emanuele 9, 12042 Pollenzo, Bra, CN Italy

**Keywords:** Herbal medicine, Ecological knowledge hybridization, Post-Soviet, Ethnomedicine, Cultural variation

## Abstract

**Background:**

While the hybridization of ecological knowledge has attracted substantial attention from researchers, the coexistence of local and allopathic medicinal traditions in literate societies widely exposed to centralized schooling and medical services has not yet been investigated. To this end, we studied the current and remembered local ethnomedical practices of Setos and neighboring Russians at the border with Estonia.

**Methods:**

During 2018–2019, we carried out 62 semi-structured interviews in the Pechorsky District of Pskov Oblast, NW Russia. For cross-border comparison, we utilized the data from 71 interviews carried out at the same time among Setos in Estonia. The Jaccard Similarity Index and qualitative comparison were used to analyze the data.

**Results:**

The study participants mentioned 819 uses of 112 taxa belonging to 54 families. More than two-thirds of the uses (565) were quoted by 36 Russian interviewees, while the remaining third (254) were quoted by 26 Seto interviewees, with the top 3 in both groups being *Viburnum opulus*, *Rubus idaeus*, and *Plantago major*. The Seto intraethnic similarity index was lower (0.43) than the interethnic similarity in Estonia (0.52) and comparable to the interethnic similarity in Russia (0.43). Setos in Russia and local Russians rely more on wild plants (86% and 80% of medicinal plants, respectively), while Setos in Estonia and Estonians show less preference to them (63% and 61%, respectively). Nevertheless, Setos tend to source wild plants available in their gardens (33% of plants for Setos in Estonia and 38% in Russia), while Russians prefer to source them in the wild (38%).

**Conclusions:**

The preference of both groups in Russia for wild plants over cultivated and purchased plants was inspired by the overall plant literacy, access to nature, and one-to-many knowledge transfer favoring wild plants. Setos in Russia reported a narrower and more homogenous set of plants transferred vertically. However, due to atomization and the erosion of horizontal connections, there are singular plant uses among Setos that overlap with the local Russian set of medicinal plants and differ qualitatively from that of Setos in Estonia.

## Background

The combination of traditional medicinal knowledge with that belonging to allopathic medicine is called hybridization, and it is characteristic of contemporary indigenous societies exposed to Western medicine and education systems. In fact, in such cases pharmaceuticals do not permanently replace traditional remedies but rather the two systems coexist [1]; moreover, the hybridization of traditional local knowledge can be a precursor to its resilience [2]. Studies on post-Soviet materials reveal that while the return to complementary and alternative medicine (CAM), to which local ecological knowledge (LEK) can be attributed, can be linked to a collapse of allopathic medicine [3], the restored accessibility to allopathic or mainstream medicine does not necessarily mean abandonment of CAM [4]. However, since traditional medicinal systems are more pragmatic, by their nature, and much less loaded with value and symbolic meaning than traditional food, they are more susceptible to change [5, 6]. Being an adaptive system, local ecological knowledge (LEK) is itself defined by a number of socioeconomic factors starting with age, gender, education, and religion, but also including income, access to a home garden, wild resources, and access to health providers, their attitude toward nature and the quality of services provided by them [7–12]. Lastly, a role in traditional knowledge preservation is played by the horizontal connections within the local community [13].

Education and schooling are usually regarded as a factor in the erosion of local ecological knowledge (LEK). It has been widely accepted for some time that there is a negative correlation between the level of education and the preservation of medicinal LEK [14–17]. However, Beltran-Rodriguez et al. [18] found no correlation between LEK and the level of education in Mestizo communities in Mexico. Reyes-Garcia et al. [15] argued that the contextualization of the content of school programs might be crucial for LEK integrity and there are suggestions that some schooling systems, on the contrary, adapt their organization to the traditional practices [19].

Indigenous ways of life have changed drastically by the expansion of industrial and post-industrial economies. Studies in Chukotka have shown that in situations of close contact not only medicinal [20] but also food practices can change dramatically [21], and language preservation can play a key role in LEK resilience [22]; however, Krupnik and Vakhtin [23] reported that there is a slow hope regarding the preservation of key concepts of ecological knowledge during language attrition. The impact is even more obvious when one examines the changes experienced by a community that was at some point separated, became a diaspora, or was divided by a newly established border. The transformations occurring in divergent communities can highlight the changes in the environmental or socioeconomic contexts linked to them.

A newly formed state border separated the Seto community 30 years ago following the collapse of the Soviet Union. Setos are a Finno-Ugric people, with the majority of the population residing in Estonia (ca. 12,000) and only about 170 individuals living in Russia, but they have remained in close contact with Russians for centuries. Setos speak a sub-dialect of the Võro dialect of Estonian and, like Russians, belong to the Orthodox faith. The traditional occupations of Setos were agriculture and pottery. Being located in a rural area on the frontier between West and East, the Seto tradition has absorbed features of both. At different times during the twentieth century, Setos resided in the territory of the Russian Empire, independent Estonia, and the Soviet Union, being exposed to collective farming and Soviet healthcare while being schooled in Estonian.

The aims of the study were:

- to document the current ethnomedical practices in the area related to the use of plants and to conduct cross-cultural and diachronic analyses;
- to consider the factors that could influence the transmission of local ecological knowledge regarding medicinal plants and its hybridization in the study area;
- to assess changes in LEK and address their potential provenance using cross-cultural and cross-border data.

## Data and methods

### Field study

During the summers 2018–2019, we conducted 62 semi-structured interviews among Setos and Russians residing in the Pechorsky District of Pskov Oblast’, Russia. In the second year of the project, we spoke for a second time to 15 participants to discuss the details of their plant use. The demographic distribution of the study participants is shown in Table 1. We aimed to recruit participants aged 40 years or above so that they had gained sufficient experience in various plant uses linked to multiple contexts. The oldest participant was born in 1916 and the youngest in 1980. The participants were recruited through convenience sampling, i.e., we spoke to individuals available on the street and in yards of country houses, and also via the snowball method, especially the Seto informants due to their limited presence in the region. Oral informed consent was always obtained before the start of the interview and the ISE ethical guidelines were followed [24].

**Table 1:** Demography of the field study participants

	Median age	Gender	Education	Religion
Setos	66	*N* = 26 16 F/10 M	2 Secondary 11 Vocational 11 College 2 Higher	Mainly Orthodox
Russians	68	*N* = 36 27 F/9 M	1 Primary 8 Secondary 5 Vocational 12 College 10 Higher	Orthodox or atheist

During the interviews, we asked about domestic plant remedies for the most common illnesses, addressing various parts of the body (head, eyes, ears, heart, stomach) or the most common ailments (cough, female or male problems, children’s diseases), the sources of information about the uses, and the temporality of use (see details below). All interviews were conducted in Russian. Seto plant names were actively elicited from Seto informants unless they were mentioned during the interview. During the course of our interaction with the study participants, we asked them to show us around their home gardens or the forest in order to locate and collect voucher specimens. We also asked our informants for permission to collect ‘dry specimens’—small portions of homemade herbal preparations, most frequently recreational or medicinal teas. Finally, we recorded the titles of books or copied magazines and other materials that were mentioned by the informants as a source of medical information.

### Data processing

The obtained materials were transcribed and tabulated according to the following categories: informant code, local plant name, Latin plant name, used part, preparation, time of use, mode of use, emic disease name, and etic disease category. For the etic disease classification, we relied on the second edition of the International classification of primary care issued by the WHO [25]. The correspondence between emic disease terms and etic ICPC-2 disease categories is indicated in Table 2.

**Table 2:** The correspondence between ICPC-2 disease categories and the diseases recorded in the field

**Blood**				
Blood cleansing	Blood dilution	Instead of aspirin	Low hemoglobin	
**Cardiovascular**				
Atherosclerosis	Heart pain	Heart support	Leg vein problems	Spasms in blood vessels
Balancing blood pressure	Heart herb	Hemorrhoids	Low blood pressure	Strengthens blood vessels
Coronary heart disease	Heart problems	High blood pressure	Shortness of breath	Vein problems
Foot tiredness				
**Digestive**				
Anti-inflammatory	Digestion recovery after oncology treatment	Intestinal problems	Liver cleansing	Stomachache in children
Bad breath	Flatulence	Intestinal problems in children	Liver problems	Stomach problems
Bile expelling	Flatulence in children	Jaundice	Poisoning	Stomatitis in children
Colitis	Gastritis	Laxative	Stomachache	Upset stomach
Diarrhea	Helminths	Liver cirrhosis	Stomachache in babies	Intestinal infections
Diarrhea in children	High acidity			
**Ear**				
Ear pain	Noise in ears			
**Endocrine**				
Cholesterol balance	Gout	Pancreas problems	Thyroid gland problems	
Diabetes	Iodine source	Salt deposition in joints		
**Eye**				
Eye irritation	Goopy eyes	Tired eyes		
**Female Genital**				
Female problems	Menorrhagia	Women's health		
**General**				
99 diseases	CO poisoning	Good for blood	Immunity	Panacea
Aches	Cold prophylactic	Good for health	Inflammation	Sudorific
Allergy	Edema	Good for heart	Mumps	Tonic
Anti-inflammatory	Fever	Good for intestines	N/A	Vitamins in spring
Infant diseases	General health	Good for stomach	Oncology	Vitamins in winter
Cleansing	General sickness	Good for vision		
**Male Genital**				
Male diseases	Prostatitis			
**Musculoskeletal**				
Arm pain	Bursitis	Knee edema	Painkiller	Rheumatism
Arthritis	Foot pain	Knee pain	Radiculitis	Wounds
Back pain	Joint problems	Leg pain		
**Neurological**				
Analgesic	Hangover	Nerve restoring		
Finger spasms	Headache	Seizures		
**Psychological**				
Sedative	Sedative for children	Sleep improvement	Sleeplessness	
**Respiratory**				
Tonsillitis (angina)	Cold	Cough	Running nose	Sore throat
Antiseptic for cold	Cold in children	Cough in children	Sinusitis	Throat inflammation
Asthma	Congested nose	Flu		
**Skin**				
Abscessed blister	Boils	Foot dryness	Pimples	Skin irritation
Abscessed wound	Bruises	Hair growth	Pus extraction	Warts
Bath for babies	Burns	Inflammation	Skin inflammation	Wounds
Bleeding	Cuts	Insect bites		
**Urological**				
Diuretic	Female genital hypothermia (most commonly expressed as UTI)	Kidney problems	Kidney stones	Urinary tract infections

The information was then organized into use instances (UI), with one UI containing information about one plant used for a certain ailment and prepared in various ways. In the data analysis, we only included the plants used personally by the participants or by their closest relatives. All uses were ordered on a temporal scale according to the interviewees’ indications (Table 3). We relied on the self-declarations of the study participants; therefore, some of the use reports, especially in the category of ‘all time,’ may have in fact been acquired during their lifetime, but not perceived as such due to the age of our participants—60 years old on average.

**Table 3:** Time of use

Time indication	Comment
**Past**	
Past long ago	Used by parents’ generation or earlier
Abandoned in childhood	Used in childhood and abandoned
Once in adulthood	Acquired in adulthood to treat a particular problem
Abandoned recently	Used throughout life and abandoned recently, usually within the last five years
**Present**	
Adulthood	Acquired in adulthood and applied when needed, including nowadays
Only now	Acquired within the last five years
All time	Known since childhood and used throughout life

To measure similarity in the cross-border context, we applied the Jaccard Similarity Index (JI), following the methodology used in González-Tejero et al. [26]:

$${\text{JI}} = \, \left[ {{\text{C}}/\left( {{\text{A}} + {\text{B}} - {\text{C}}} \right)} \right] \, \times { 1}00,$$

where A is the number of wild plant taxa reported in sample A and B is the number of wild plant taxa reported in sample B, and C is the number of taxa common to both samples. We only included plants in the data sample that were mentioned more than three times in each ethnic group. The Jaccard similarity index is known to be biased for small samples, especially those with a high incidence of rare species [27]. To address this issue, we only included plants in the data sample that were mentioned more than three times in each ethnic group.

Venn diagrams were created using the web service provided by the Bioinformatics and Evolutionary Genomics Unit of the University of Ghent [28] and BioVenn [29].

We started our interviews with a focus on wild food plants and then proceeded to home remedies, not limited to wild plants, for the most common illnesses. These also include cultivated and purchased plants (see remarks in Table 5). Nine taxa were identified on the genus level as individual species are used interchangeably in local practice and some could not be identified: *Bergenia*, *Betula*, *Calanchoe*, *Hypericum*, *Mentha*, *Pelargonium*, *Rosa*, and *Sphagnum*. Since Matricaria chamomilla is not very common in the region and is often confused with Tripleurospermum inodorum and other plants of the Asteraceae family, we combined them into the ethnotaxon ROMASHKA (Russian for chamomile).

Romanization of the Russian language was made following the ALA-LC (American Library Association—Library of Congress) Romanization without Diacritics set of standards. Russian geographic names are provided according to their English spelling.

We used Efimov and Konechnaia [30], being the most up-to-date and comprehensive description of Pskov region flora, as a reference for plant habitats with a focus on synanthropic plants. However, we updated their data in several cases when wild plants were pointed out by our interviewees in their gardens or immediately next to their plots.

The voucher specimens are deposited at the Komarov Botanical Institute of the Russian Academy of Sciences in Saint Petersburg and are available online at [31] bearing the following codes: LE 01063392-461, LE 01063463, LE 01063465, LE 01063466, LE 01063469, LE 01063477, LE 01063496, LE 01063498, LE 01063504-6, LE 01063510-14, LE 01063544, LE 01063578, and LE 01063946. The dry specimens bearing the codes dsPCH19-001-032 are stored in the first author’s personal archive. The plant nomenclature followed the World Flora Online database [32] and Flora Europaea [33]; the plant families were classified according to the Angiosperm Phylogeny database [34].

## Research area

**Fig. 1:**
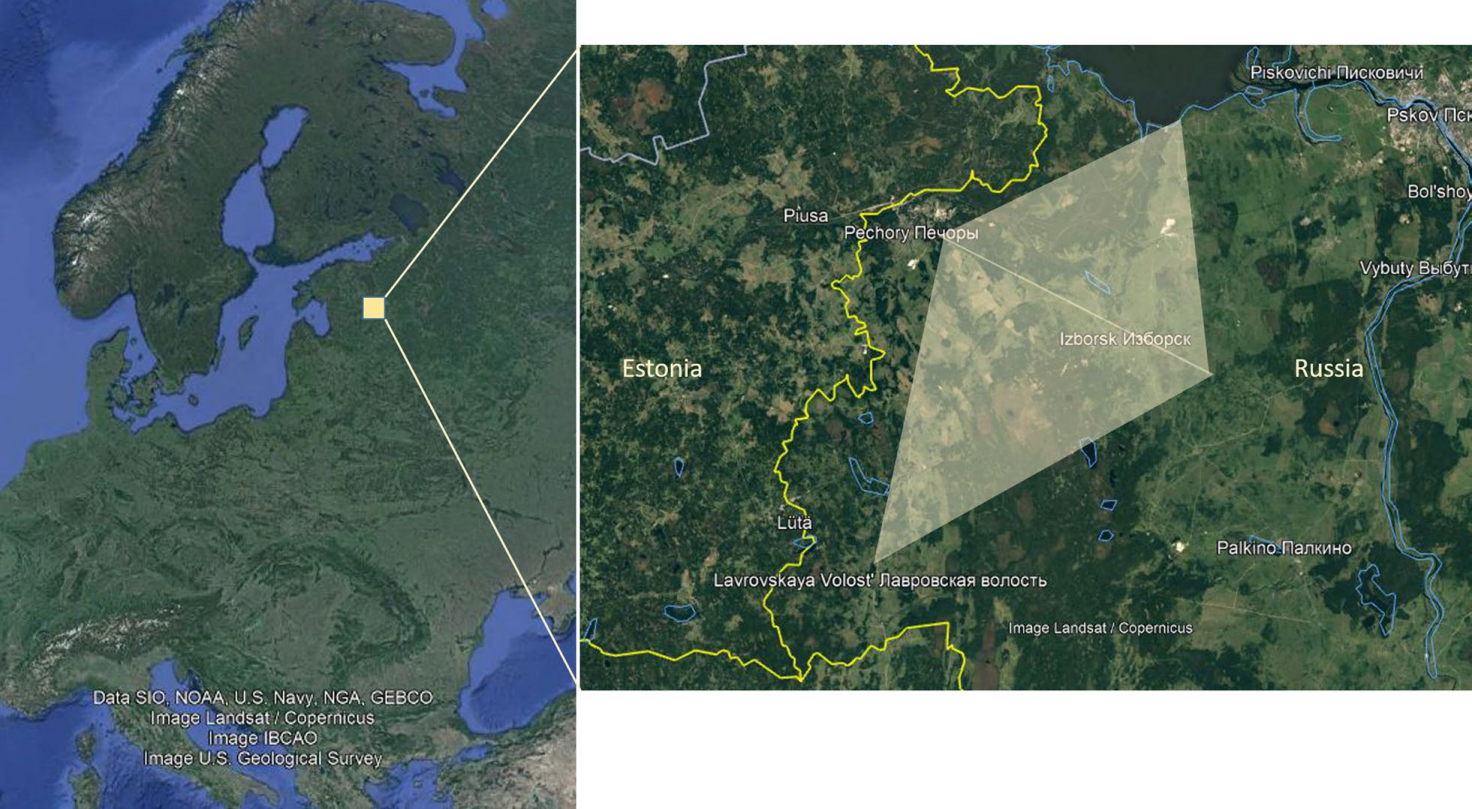
The research area on the border of Estonia and the Russian Federation

Pechorsky District (1251 km^2^) is a subdivision of Pskov Oblast located in northwestern Russia on the border with Estonia (Fig. 1). The study area lies between Lake Peipus (3550 m^2^) to the north and the Haanja Upland to the south, with an elevation up to 200 m. Depressions are frequently transformed into wetlands which often become bogs rich in peat. The Izborsk area of Pechorsky is characterized by the peculiar karst topography. The soils are ash gray (podzol). The region does not feature great rivers, so all local communication is conducted over land. Some of the roads are subject to seasonal flooding rendering some areas virtually inaccessible, especially in autumn and spring. Pechory is the cultural, spiritual, and administrative center of the region, while other prominent settlements coinciding with historical parishes include Izborsk (also an important cultural, ecological, and tourist site), Panikovichi, Lavry, Zales’ye, Podles’ye, and Krupp. The majority of Setos have moved from their family homes at least once since 1940 (reasons varying from forced relocation to Siberia, to emigration to Estonia, to moving from abandoned villages and urbanization). The local population of the region is retired or close to retirement age. The main occupation in the region is farming. Some individuals seek jobs in Estonia (e.g., electrician, truck driver) or next to the border (e.g., customs officer). One of the major employers in the region is Evrokeramika, a manufacturing company producing ceramic tiles. The younger inhabitants have arrived in the region within the last two decades seeking a ‘more healthy’ rural environment for their families and an occupation like farming or traditional crafts.

### Vegetation of Pechorsky District

The environment of the Russian part of Setomaa had long been affected by human activities until several decades ago when the majority of agricultural lands were abandoned due to economic instability. While Pechorsky rayon, as part of Pskov Governorate, was described in several naturalists’ reviews [35], the first detailed study dates back to 1928 [36]. According to that investigation, the studied area had suffered from deforestation: Forests were less than 50 years old, mostly consisting of pine forests on infertile lands and tiny patches of spruce-birch forests on fertile ones. The majority of lands were arable or semi-natural meadows [36]. The second thorough study of the region documented the abandonment of agricultural lands and the continued low percentage of forests [37]. Between 1990 and 2000, arable lands diminished from 77 to 60%, while natural meadows located around bodies of water, representing 14% of arable land, started to be succeeded by shrubs. The forests still occupied a bit less than 1/5 of the area, and consisted mainly of pines (82%) and, to a much lesser extent, birch, and spruce (8.5% and 8%, respectively). Settlements demonstrated a rapid growth from 0.6% to 14%; meanwhile, biodiversity was in gradual decline [37]. Currently, there are 1248 vascular plant species in Pskov region [30].

### The ‘discovery’ of Setos and early contact with Russians

The presence of Finnic tribes around the Baltic Sea dates back to 3500 years ago [38]. Setos occupied the territory adjacent to Lake Peipus, Lake Pskov, and the Velikaya River [39]. In 862, East Slavs invaded this territory and built the fortress of Izborsk attached to the Principality of Pskov. The creation of Pskov-Pechory Monastery in 1473 occurred shortly before the annex of the independent Principality of Pskov to the Russian state, which fostered the Orthodox faith. Setos did not understand the Orthodox sermons delivered in Old Slavonic, and at the same time their culture preserved archaic pagan as well as catholic features [40–42].

The earliest mention of ‘Pskov Estonians’ appeared in the eighteenth century, but the first comprehensive descriptions were made only in the late nineteenth century by linguist Yuri Trusman (1856–1930) and folklorist Jakob Hurt (1839–1906). While initial researchers were puzzled by the combination of ‘Estonian’ language, Orthodox faith, distinctive female clothing, and celebrations, later studies also specified an open hearth and dwellings combining the barn and living space under one roof [43, 44].

There are no published records regarding the historical herbal medicines of Setos from the nineteenth century, just a few notes concerning plant use sent by correspondents of Estonian pastor and folklorist Jakob Hurt (1839–1906) in 1889 and 1904, and a dozen records collected by Hurt himself during his Seto expedition conducted in 1903 [45]. In the 1930s, the first Estonian ethnobotanist Gustav Vilbaste (1885–1967) collected, with the help of students of several Setomaa schools, a comparable body of knowledge [45] which we will use for diachronic analysis.

### School education in Pechorsky District, emigration, and the disintegration of the Seto community in Russia

Since the end of the nineteenth century, Setos have been exposed to the waves of Russification and Estonianization. Primary education became widely available in Pechory District after 1864 and was provided in parochial schools (focused on scripture) as well as in locally governed zemstvo schools teaching the Russian language and mathematics. There were seven schools as of 1890, and 20 soon after 1900 [46]. In 1891, the Russian clergy in Pskov raised the question of leading sermons and establishing education in a language ‘understandable to them’ so that they could be followed by Setos [47]; however, the final goal of such schools was Russification of the non-Russian locals. In 1892, a parochial school in the village of Avichishche (now Obinitsa, Estonia) was established. In 1899, it provided education to 66 Seto pupils among another 110 [46]. However, compared to Livonia, where parochial education had been widespread since 1840, the number of literate Seto and Russian children (especially girls) was low.

During the Estonian period (1920–1940), education in Estonian was made available in a systematic manner as part of the Estonianization policy. The Seto people were given family names (surnames) which they did not have before. Petseri (Estonian name of Pechory) gymnasium opened in 1919, replacing a primary school founded by Orthodox priest Father Karp in 1905, which offered education in Estonian as well as in Russian [46]. The first reading book in Seto was published in 1922. Church congregations were also segregated: Ethnic Russians were allowed to preach in Russian, and then Seto congregations began to deliver sermons in Estonian [48]. After Pechorsky District was annexed by the USSR, the network of primary schools with education in Estonian was preserved. However, in the 1970s, due to massive centralization, schools in the less populated Troitskoe and other villages were closed. Pechory gymnasium, converted into School № 2 (later Pechory linguistic gymnasium), reopened in 1957 and started to operate as a boarding school providing secondary education in Estonian to all the Setos of the district. Those closer to the Estonian border could attend the school in Luhamaa up until 1992 when the border regime with Estonia was introduced.

The lack of vocational or higher education in the Estonian language was one of the main reasons for the mass immigration of Setos into the territory of Estonian SSR in the 1950s. In the 2004–2005 academic year, due to the lack of Seto pupils, the Pechory linguistic gymnasium ceased teaching in Estonian and converted to Russian [49]. Currently, it only offers extracurricular activities in Estonian and Seto in the framework of a Seto ethnocultural center: Tsirgukõsõ (Seto ‘birdies’) choir, culinary and other exhibitions, and masterclasses.

The current education system in the Russian Federation mainly follows that of the former Soviet Union. Education is divided into three levels: primary, secondary, and higher education. Primary education consists of the first three or four years of school. To complete secondary education, one should either graduate after 10 or 11 years of schooling or complete 8 or 9 years and then graduate from a vocational school, having become a qualified worker or technician. After that, one can enter higher education, attending either college (or institute) to become a pedagogical worker or technical specialist, or university to acquire a scientific qualification.

There are no statistics regarding the language situation of the Seto language, as it is not separated by linguists from the Southern Estonian dialect Võro. Currently, there are about 170 Setos in Pechorsky District (Russian census 2010) and about 5000 across the border in Estonia [50]. Our field observations as well as the presumptions of linguists [50] indicate that Seto is spoken mainly by middle-aged or older individuals, while all speakers are bilingual in Russian and/or Estonian depending on their place of residence. In Estonia, education in Seto is offered on an extracurricular basis. The language is predominantly used in the private sphere as well as in events dedicated to Seto culture and *leelo* (Seto polyphonic singing).

The totality of the cultural and economic processes of the twentieth century led to the fragmentation of the Seto community on the Russian side of the border. Some of the important factors included the system of isolated farmsteads, the system of ‘social lift,’ and later the economic disparity that provoked massive emigration from Pechory District to Estonia and to regional Russian centers, and, on the other side, the influx of qualified workers recruited from Pskov and Leningrad and later the incoming summer folks from Moscow and Saint Petersburg (see [43]). From our 26 interviews, only two featured families in which both spouses were Seto. Due to geographical isolation—scarce population spread across isolated farmsteads—the majority of Setos have a chance to meet each other only at celebrations organized on both sides of the border and dedicated to the Kingdom of Seto (Estonia, July–August) and family meetings (Rus. *Semeinye vstrechi*, last week of August, Russia).

### Allopathic medicine of the region

In this article, the dichotomy between allopathic medicine and ethnomedicine as part of LEK will be used. The terms ‘Western medicine’ and ‘biomedicine’ do not particularly fit the case of the medical practices in the healthcare system of the Soviet Union, as it sometimes utilized the approaches of various traditional non-western medicines, such as Tibetan medicine.

The healthcare infrastructure of Pechory District consists of Pechory Hospital (founded 1885) and eight GP offices in Izborsk, Lavry, Lazarevo, Mikovitsy, Novoizborsk, Panikovichi, Pechki, and Rotovo. The hospital is equipped with 53 beds offering surgical, gynecological, neurological, pediatric, and general therapeutic care. The healthcare facility also includes a clinic, an obstetrics center, a pediatric center, and an emergency unit with two crews and a dentist’s room. All six pharmacies of Pechory District are located in Pechory.

Throughout the Soviet era, one of the main goals of local healthcare providers was to medicalize pregnancy, that is, the delivery taking place in the hospital or at least being assisted by a qualified medical worker in case of home delivery. While the current infant mortality rate in the region is 4 per 100,000, in 1959 it was as high as 40.6 per 1000 (or, according to other computations, 46.4; see [51, p. 6]).

Basic medical services as well as medication for certain patient categories are now covered by the state, but due to high demand certain specialists and drugs are sometimes inaccessible. The compulsory medical insurance offered by the state to all citizens and foreign visitors covers the GP, specialized medical assistance, as well as emergency hospitalization. Although medicines are currently provided for free to some social groups (disabled people, infants of up to three years old, war veterans, etc.) and to certain patients (those with diabetes, tuberculosis, etc.), they can be inaccessible due to shortages caused by various factors [52, 53]. Some of the shortages involve the replacement of imported drugs, changes in pharmaceutical certification, as well as insufficient financing of local healthcare providers. Nevertheless, 70% of prescription medicines are eventually paid for by the end consumer [54], which makes the problem of limited accessibility to prescription drugs due to high pharmaceutical costs quite significant.

The majority of the study participants were retired people whose allowance was not compatible with purchasing medicaments, one pack of which could cost up to one twentieth of the average monthly retirement allowance. Even though for some individuals the pharmaceuticals are subsidized, the poor road and transport network becomes an obstacle. Most older participants did not own a car, which made access to the nearest pharmacies in Pechory extremely time-consuming, and in the case of decreased mobility nearly impossible, given the schedule and penetration of the public transportation network. The combination of these factors has made them dependent on their children for sourcing and purchasing the needed pharmaceuticals, and has encouraged the use of traditional ‘self-care’ practices [55].

## Results

### General overview

The study participants mentioned 738 uses of 106 taxa belonging to 50 families (Table 4, differing elements are underlined). More than two-thirds of the uses (484) were recorded among the 36 Russian participants, while the remaining third (254) were recorded among the 26 Seto participants. Overall, Setos used 67 taxa, with every person mentioning 8.2 taxa on average (standard deviation 5.61), whereas Russians used 101 taxa, with an average of 11 taxa per person (standard deviation 8.64).

**Table 4:** Cross-cultural distribution of the most important plant families, taxa, and disease categories. Differing elements are underlined

	Setos, *N* = 26	Russians, *N* = 36
Families	Asteraceae (45 UI) Ericaceae (39) Rosaceae (34) Adoxaceae (19) Plantaginaceae (14)	Asteraceae (107 UI) Rosaceae (84) Ericaceae (55) Lamiaceae (34) Plantaginaceae (29)
Taxa	*Viburnum opulus* L. (19) *Rubus idaeus* L. (14) *Plantago major* L. (14) *Vaccinium oxycoccos* L. (13) *Hypericum perforatum* L. (12)	*Rubus idaeus* L. (34) *Plantago major* L. (29) *Viburnum opulus* L. (25) *Hypericum perforatum* L. (22) *Vaccinium myrtillus* L. (21)
Disease categories	Respiratory (66) General (46) Cardiovascular (29) Digestive (28)	Respiratory (109) Digestive (100) General (78) Skin (72)

The detailed list of local medicinal plant uses with local names and voucher specimen numbers is presented in Table 5. This table lists use instances (UI), with each UI referring to a use of a certain plant part for a specific condition, whatever the preparation.

**Table 5:** Plants and their medicinal uses recorded during the fieldwork

Family/taxon, voucher specimen, local names/part	Medicinal use	Mode of use	Russians, UI *N* = 36		Setos, UI *N* = 26	
			Past	Present	Past	Present
Acoraceae			**1**			
*Acorus calamus* L.	RU *puchka*		**1**			
Stalk bottom	Iodine source	Raw	**1**			
Adoxaceae			**8**	**17**	**12**	**7**
*Viburnum opulus* L.* LE 01063405	RU *kalina*	S lodjapuu, *kalina* (RU)	**8**	**17**	**12**	**7**
Flowers	Cough	Infusion	1			
Fruit	Antiseptic for cold	Raw		1		
	Cold	Frozen, infusion, jam, juice, raw	2	3	7	3
	Cough	Frozen, infusion, jam, juice, raw	3	3		
	Fever	Raw		1		
	Good for the heart	Infusion				1
	High blood pressure	Frozen, infusion, jam, juice, raw	1	3	1	2
	Heart problems	Frozen, jam, raw		1	1	1
	Heart support	Raw jam or infusion		1		
	Immunity	Jam			1	
	Medicine	Dried	1			
	Shortness of breath	Raw			1	
	Spasms in blood vessels	Frozen		1		
	Strengthens the blood vessels	Frozen		1		
	Vitamins in spring	Raw		1		
Leaves	Heart support	Infusion		1		
Twigs, bark	Cold	Infusion			1	
Amaranthaceae			**1**			
*Beta vulgaris* L.^†^	RU *svekla kormovaia*		**1**			
Leaves	Wounds	Raw, topical application	1			
Amaryllidaceae			**2**	**3**	**2**	
*Allium cepa* L.^†^	RU *luk*	S *luk* (RU)	**2**	**3**	**2**	
Bulbs	Cold	Boiled in milk			2	
	Cough	Baked	1			
	Pus extraction, boils	Baked, topical application	1	1		
Peels	Blood cleansing	Infusion		1		
	Cough			1		
Apiaceae			**3**	**13**	**1**	**1**
*Anethum graveolens* L.^†^ LE 01063460	RU *ukrop*	S *ukrop* (RU)	**3**			**1**
Seeds	Flatulence or stomachache in babies	Decoction or infusion	3			1
*Carum carvi* L.* dsPCH19-010	RU *tmin*	S *tmin* (RU)		**5**	**1**	
Seeds	Good for health	Infusion		1		
	Heart problems			1		
	Intestinal problems or flatulence			1	1	
	Liver problems			1		
	Stomach problems			1		
*Daucus carota* subsp. *sativus* (Hoffm.) Arcang.^†^	RU *morkov’*			**4**		
Aerial parts	Good for the blood	Infusion		1		
	Good for health			1		
	Salt deposition in joints			1		
Root	Pus extraction	Raw		1		
*Levisticum officinale* W.D.J.Koch^†^	RU *liubistok*			**2**		
Aerial parts	Good for health	Raw		1		
	Heart problems	Raw		1		
*Petroselinum crispum* (Mill.) Fuss^†^	RU *petrushka*			**2**		
Aerial parts	Kidney problems	Infusion		1		
	Liver problems			1		
Asphodelaceae			**2**	**5**		**1**
*Aloe arborescens* Mill.^†^	RU *aloe, stoletnik*	S *aloe* (RU)	**2**	**5**		**1**
Aerial parts	Tonic	Tincture		1		
Juice	Abscessed blister or wound	Topical application		2		
	Boils			1		
	Eye irritation	Drops		1		
	Running nose		2			1
Asteraceae			**48**	**59**	**33**	**12**
*Achillea millefolium* L. LE 01063441, LE 01063544, dsPCH19-004, dsPCH19-006	RU *tysiachelistnik, tysiachelistvennik*	S *tysiahelistnik* (RU), raudrohi, verehaine	**6**	**6**	**5**	
Aerial parts	Bleeding	juice, topical application	1			
	Diarrhea	Infusion	2	1		
	Diarrhea in children		1			
	Good for health			1	1	
	High blood pressure			1		
	Low hemoglobin			1		
	Stomachache			1		
	Upset stomach		1			
	Wounds	Topical application			1	
Flowers	High acidity in the stomach	Infusion		1		
	Wounds	Juice, topical application	1			
Leaves	Cuts	Topical application			1	
	Intestinal problems in children	Infusion			1	
	Wounds	Topical application			1	
*Arctium tomentosum* Mill.	RU *lopukh, repeinik*	S *lopukh, dedovnik(i)* (RU)	**7**	**12**	**5**	**2**
Leaves	Arthritis	Topical application		1		
	Foot dryness			1		
	Foot pain			1		
	Foot tiredness			1		
	Gout			1		
	Hair growth		1			
	Headache		1	1		
	Joint problems		3		3	1
	Knee edema			1		
	Knee pain			4		1
	Leg vein problems		1			
	Painkiller				1	
	Female problems	Tincture			1	
	Gout	Decoction: topical application	1			
	Knee pain	Tincture		1		
*Artemisia absinthium* L. dsPCH19-031	RU *polyn’*	S *polyn’* (RU)	**2**	**2**	**2**	
Aerial parts	Good for health	Decoction	1			
	Sedative	Infusion	1			
	Stomachache				1	
	Stomachache in children				1	
	Stomach problems	Tincture		1		
	Helminths	Decoction		1		
*Bidens tripartita* L.	RU *chereda*	S *chereda* (RU)	**8**		**2**	
Aerial parts	Allergy	Infusion	1			
	Bath for babies	Decoction, infusion	6		2	
	Pimples	Infusion	1			
*Calendula officinalis* L.^†^ LE 01063421, dsPCH19-028	RU *nogotki, kalendula*	S *kalendula* (RU)	**2**	**6**		**3**
Aerial parts	Heart problems	Infusion				1
Flowers	Antiseptic for cold	Infusion, tincture		3		
	Cold	Infusion		1		
	Cuts	Topical application	1			
	Liver cleansing	Infusion		1		
	Liver problems					2
	Sleeplessness			1		
	Warts	Topical application	1			
*Chamaemelum nobile* (L.) All.^†^		S *romashka sadovaia* (RU)				**1**
Aerial parts	Heart problems	Infusion				1
*Cichorium intybus* L.	RU *tsikorii*	S *tsikorii* (RU)		**2**	**1**	
Root	Liver problems, cirrhosis	Infusion		2		
	Medicine				1	
*Comarum palustre* L.	RU *sabel’nik*		**1**	**2**		
aerial parts	Joint problems	Tincture: topical application or oral administration		2		
root	Joint problems	Tincture: topical application	1			
*Cota tinctoria* (L.) J.Gay LE 01063394	RU *zheltaia romashka*		**3**	**1**		
Aerial parts	Good for health	Infusion	1			
	Heart problems			1		
	Jaundice		1			
	Liver problems		1			
*Helichrysum arenarium* (L.) Moench dsPCH19-002	RU *bessmertnik*			**1**		
Aerial parts	Liver problems	Infusion		1		
*Inula helenium* L. *	RU *deviasil*			**2**		
Root	Cough	Tincture		1		
	Stomach problems			1		
*Matricaria chamomilla* L.* dsPCH19-023 *Tripleurospermum inodorum* (L.) Sch.Bip. LE 01063445, LE 01063422, dsPCH19-012	RU *romashka (aptechnaia)*	S *romashka* (RU), teekummel, karikakar	**3**	**7**	**4**	**4**
Aerial parts	Bath for babies	Decoction, infusion: bath	1	1	1	
	Cold	Infusion			1	1
	Cold in children			1		
	Colitis			1		
	Cough		1			1
	Good for health				1	
	Heart problems					1
	Medicine		1			
	Painkiller					1
	Stomach problems			4		
	Upset stomach				1	
*Matricaria discoidea* DC. LE 01063395, LE 01063416, LE 01063444, dsPCH19-011, dsPCH19-019	RU *romashka, aptechnaia romashka, romashka ulichnaia*	S *romashka (ulichnaia)* (RU), teekummel	**9**	**5**	**8**	
Aerial parts	Anti-inflammatory	Infusion		1		
	Bath for babies	Decoction, infusion: bath	3	2	2	
	Cold	Infusion	1	1	3	
	Cough		2	1	1	
	Eye irritation	Decoction, drops			1	
	Fever	Infusion	1			
	Mumps	Parboiled, topical application			1	
	Sore throat	Infusion, rinse	1			
	Stomatitis in children		1			
*Seriphidium cinum* (Berg ex Poljakov) Poljakov^‡^	RU *tsitvarnoe semia*		**1**			
Seeds	Helminths	Infusion	1			
*Tanacetum vulgare* L. LE 01063442, LE 01063402	RU *pizhma, riabinka*	S *pizhma* (RU), ussiroht	**2**		**1**	
Aerial parts	Diarrhea	Infusion	1			
	Helminths		1			
Inflorescences	Helminths	Infusion			1	
*Taraxacum officinale* (L.) Weber ex F.H.Wigg. LE 01063407	RU *oduvanchik*	S *oduvanchik* (RU)	**1**	**3**		**1**
Flowers	Immunity	Jam		1		
	Joint problems	Tincture: topical application		1		
Root	Liver problems	Infusion		1		
	Pancreas problems	Tincture				1
Latex	Warts	Raw	1			
*Tussilago farfara* L. LE 01063452	RU *mat’-i-machekha*	S *mat’-i-machekha* (RU), paiseleht	**3**	**10**	**5**	**1**
Aerial parts	Cold	Infusion			1	
	Cold in children				1	
	Cough		2	8		
Leaves	Cough in children	Infusion		1		
	Fever					1
	Good for health				1	
	Joint problems	topical application			2	
	Leg vein problems		1			
	Wounds			1		
Betulaceae			**3**	**6**	**1**	
*Alnus incana* (L.) Moench, *Alnus glutinosa* (L.) Gaertn	RU *ol’kha*	S *ol’kha* (RU)	**2**	**1**	**1**	
Female catkins	Diarrhea	Infusion	1			
	Poisoning		1			
	Stomach problems			1		
Young leaves	Wounds	Topical application			1	
*Betula* sp. LE 01063453 (incl. *Betula pendula* Roth, *Betula pubescens* Ehrh.)	RU *bereza*		**1**	**5**		
Buds	Diuretic	Infusion		1		
	Good for health			1		
	Oncology			1		
Leaves	Good for health	Infusion		1		
Sap	Cleansing	Drink		1		
Twigs	Joint problems	Steam bath whisk	1			
Brassicaceae			**9**	**11**	**3**	**8**
*Brassica oleracea* L.^†^	RU *kapusta*	S *kapusta* (RU)	**7**	**7**	**2**	**7**
Leaves	Arm pain	Topical application			1	
	Edema					1
	Gout			1		
	Hangover	Brine from lactofermentation		1		
	Headache	Topical application	4	2		2
	Joint problems		3	1	1	1
	Knee pain			1		1
	Seizures					1
	Skin inflammation			1		1
*Capsella bursa-pastoris* (L.) Medik. LE 01063432, LE 01063427	RU *pastush’ia sumka*	S *pastush’ia sumka* (RU)	**1**		**1**	
Aerial parts	Gastritis	Infusion	1			
	Menorrhagia				1	
*Raphanus raphanistrum* subsp. *sativus* (L.) Domin^†^	RU *red’ka (chernaia)*	S *red’ka* (RU)	**1**	**4**		**1**
Root	Cold	Juice		2		1
	Cough		1	1		
	Tonsillitis			1		
Cannabaceae					**1**	
*Humulus lupulus* L.* LE 01063406		S *khmel’* (RU)			**1**	
Hops	Hair growth	Decoction			1	
Caprifoliaceae			**7**	**5**	**7**	
*Valeriana officinalis* L.	RU *valer’iana, valer’ianka, valer’ianovka*	S *valer’iana* (RU)*,* paldõrjan	**7**	**5**	**7**	
Flowers	Headache	Infusion		1		
Root	Asthma	Infusion	1			
	Coronary heart disease	Tincture		1		
	Good for the heart	Infusion	1			
	Heart problems	Tincture	1		1	
	High blood pressure	Infusion, tincture	1	1		
	Radiculitis	Tincture, topical application			2	
	Sedative	Infusion, tincture	3	2	2	
	Sedative for children	Tincture			2	
Caryophyllaceae			**2**			
*Silene vulgaris* (Moench) Garcke LE 01063400	RU *belye fonariki*		**1**			
Aerial parts	Diarrhea	Decoction	1			
*Stellaria media* (L.) Vill. LE 01063424	RU *mokritsa*		**1**			
Aerial parts	Joint problems	Topical application	1			
Commelinaceae				**3**		
*Callisia fragrans* (Lindl.) Woodson^†^ LE 01063401	RU *zolotoi us*			**3**		
Aerial parts	Joint problems	Tincture		1		
	Insect bites			1		
Shoots	Bursitis	Tincture		1		
Crassulaceae						**1**
*Calanchoe* sp.^†^		S *kalankhoe* (RU)				**1**
Juice	Running nose	Drops				**1**
Cucurbitaceae				**1**		
*Cucumis sativus* L.^†^	RU *ogurtsy*			**1**		
Fruit	Hangover	Brine from lactofermentation		**1**		
Cupressaceae			**2**	**2**	**6**	**1**
*Juniperus communis* L. LE 01063408	RU *veres, mozhzhevel’nik*	S *veres* (RU), *veresk* (RU), *mozhzhevel’nik* (RU), kadaja, kadakas, katai	**2**	**2**	**6**	**1**
Fruit	Cold	Infusion		1	1	
	Good for health				1	
Twigs	Aches	Steam bath whisk			1	
	Back pain		1		1	
	Cold	Infusion		1		
	Joint problems	Steam bath whisk	1		2	
	Leg vein problems	Decoction				1
Dennstaedtiaceae			**1**	**1**		
*Pteridium aquilinum* (L.) Kuhn	RU *paporotnik trekhlistnyi*		**1**	**1**		
Leaves	Headache	Put under headscarf while in forest	1			
	Sleep improvement	Put in mattress or pillow		1		
Dioscoreaceae				**1**		
*Dioscorea communis* (L.) Caddick & Wilkin^‡^	RU *kremlevskaia trava*			**1**		
Aerial parts	Thyroid gland problems	Tea		**1**		
Dryopteridaceae				**2**	**1**	
*Dryopteris filix-mas* (L.) Schott	RU *paporotnik*	S *paporotnik* (RU)		**2**	**1**	
Aerial parts	Back pain	Dried, put in mattress		1		
	Knee pain	Topical application			1	
	Sleep improvement	Dried, put in mattress		1		
Elaeagnaceae				**3**		
*Elaeagnus rhamnoides* (L.) A.Nelson *	RU *oblepikha*			**3**		
Faded autumn leaves	Finger spasms	Infusion		**1**		
Leaves	Good for health	Infusion		**2**		
Equisetaceae			**1**	**5**		
*Equisetum arvense* L. LE 01063431	RU *khvoshch, pupyshi*		**1**	**5**		
Aerial parts	Diarrhea	Infusion	1			
	Diuretic			1		
	Kidney problems			1		
	Low hemoglobin			1		
	Prostatitis			1		
Spring shoots	Good for health	Raw		**1**		
Ericaceae			**11**	**44**	**11**	**28**
*Arctostaphylos uva-ursi* (L.) Spreng. LE 01063410	RU *toloknianka*	S *toloknianka, medvezh’i ushki* (RU)		**7**		**3**
Aerial parts (incl. fruit), leaves	Diuretic	Infusion		4		1
	Heart problems			1		
	Kidney stones					1
	Kidney problems			1		1
	Leg vein problems			1		
*Calluna vulgaris* (L.) Hull LE 01063447, dsPCH19-005	RU *elochki*	S kanarbik		**1**	**1**	
Aerial parts	Cough	Infusion			1	
	Heart problems			1		
*Ledum palustre* L. LE 01063438	RU *bagul’nik*	S *bagul’nik* (RU)		**2**		**2**
Aerial parts	Cold	Infusion				1
	Cough			2		
	Shortness of breath	Decoction				1
*Vaccinium myrtillus* L. LE 01063440, dsPCH19-025	RU *chernika*	S *chernika* (RU), mustikas, must’kas	**4**	**17**	**4**	**8**
Aerial parts (with fruit)	Diarrhea	Infusion				1
	Diarrhea in children			1		
	Diuretic			1		
	Good for health			1		
	Good for vision					1
	Heart problems			1		
Fruit	Diabetes	Raw		1		
	Diarrhea	dried, decoction	2		3	1
	Diarrhea in children	Dried		1		
	Good for vision	Dried, jam, kissel, raw, raw jam		9	1	4
	Stomach problems	Dried	1	2		
	Tired eyes	Raw				1
	Upset stomach	Dried	1			
*Vaccinium oxycoccos* L. LE 01063435	RU *kliukva*	S *kliukva* (RU), kuremari	**4**	**7**	**4**	**9**
Fruit	Balancing blood pressure	Macerated		1		
	Blood cleansing	Macerated, raw				1
	CO poisoning	Topical application (ears)	1		1	
	Cold	Macerated, raw			1	1
	Cough					1
	Ear pain	Topical application	1			
	Eye irritation	Drops	1			
	Fever	Decoction, macerated, raw	1	3	2	2
	Headache	Raw		1		
	Heart problems	Decoction, raw		1		2
	High blood pressure	Raw				2
	Instead of aspirin	Decoction		1		
*Vaccinium uliginosum* L. LE 01063439		S *golubika* (RU)				**1**
Fruit	Good for vision	Raw				1
*Vaccinium vitis-idaea* L. LE 01063412	RU *brusnika* (fruit, plant), *brusnichnik* (aerial parts)	S *brusnika* (RU), palohkas	**3**	**10**	**2**	**5**
Aerial parts	Diuretic	Infusion	1	2		1
	Kidney problems		2	2		
Fruit	Cold	Macerated				1
	Diuretic	Raw		1		
	Heart problems	Macerated, raw		2		
	Kidney problems	Macerated				1
Leaves	Cold	Infusion			1	
	Diuretic			1		2
	Good for health				1	
	Leg vein problems			1		
	Urinary tract infections			1		
Fabaceae			**1**	**5**		**1**
*Trifolium montanum* L. LE 01063393, dsPCH19-009, dsPCH19-021	RU *belyi klever*			**1**		
Aerial parts	Stomachache	Infusion		1		
*Trifolium pratense* L. LE 01063455, LE 01063456, dsPCH19-024	RU *klever krasnyi*	S *klever krasnyi* (RU)	**1**	**4**		**1**
Aerial parts	Heart problems	Infusion				1
Inflorescences	Atherosclerosis	Tincture		1		
	Heart support	Infusion		1		
	Medicine	Tincture	1			
	Sleep improvement	Dry, put in mattress		1		
Fagaceae			**1**	**2**	**3**	
*Quercus robur* L. LE 01063451	RU *dub, zheludi* (fruits)	S *dub* (RU)	**1**	**2**	**3**	
Bark	Diarrhea	Infusion		2	1	
	Stomachache	Decoction			1	
	Stomach problems	Infusion			1	
Fruit	Diarrhea	Baked	1			
Geraniaceae				**3**	**1**	**1**
*Pelargonium* sp.^†^	RU *geran’ (pakhuchaia)*	S *geran’* (RU)		**3**	**1**	**1**
Leaves	Ear pain	Topical application		3	1	
	Noise in ears					1
Grossulariaceae			**2**	**3**	**4**	
*Ribes nigrum* L. *	RU *chernaia smorodina*	S *chernaia smorodina* (RU)	**2**	**3**	**4**	
Fruit	Cold	Jam infusion			1	
Leaves	Cold	Infusion		1	1	
	Congested nose				1	
	Cough				1	
	Headache		1			
	High blood pressure		1			
Twigs	Anti-inflammatory	Infusion		1		
	Vitamins in winter			1		
Hypericaceae			**8**	**14**	**4**	**8**
*Hypericum* spp*. (including H. perforatum* L. LE 01063443, LE 01063428, dsPCH19-007, dsPCH19-018)	RU *zveroboi*	S *zveroboi* (RU), naistepuna	**8**	**14**	**4**	**8**
Aerial parts	Abdominal infections	Infusion		1		
	Cold		1	2	1	
	Diarrhea			1		
	Female genital hypothermia (UTI)				1	
	Female problems					1
	Good for health		1	1	2	3
	Good for the intestines		1			
	Good for the stomach		1			
	Heart problems		1	2		2
	Inflammation			1		1
	Knee pain	Oil extract				1
	Liver problems	Infusion	1	1		
	Panacea, ‘99 diseases’		1	2		
	Sedative			1		
	Stomach problems		1	1		
	Upset stomach			1		
Lamiaceae			**6**	**28**	**1**	**10**
*Leonurus quinquelobatus* Gilib. LE 01079359, LE 01063471	RU *pustyrnik*	S *pustyrnik* (RU)	**1**	**7**		**1**
Aerial parts	Heart pain	Tincture		1		
	Heart herb			1		
	Heart problems	Infusion, tincture		2		
	High blood pressure			3		
	Sedative	Infusion	1			1
*Mentha aquatica* L.	RU *miata rechnaia*			**1**		
Aerial parts	Good for health	Infusion		1		
Mentha sp.* dsPCH19-001, dsPCH19-029 *Mentha arvensis* L. LE 01063473 *Mentha longifolia* (L.) Huds. LE 01063506, LE 01063463, LE 01063465, LE 01063504 *Mentha piperita* L. LE 01063474 *Mentha spicata* L. LE 01063461 *Mentha x piperita x longifolia* LE 01063505	RU *miata*	S *miata* (RU), mjatad, münt		**9**	**1**	**4**
Aerial parts	Cold, flu	Infusion		3		
	Heart problems					1
	Sedative			3		3
	Sudorific			2		
Leaves	Bad breath	Raw, chewing			1	
	Liver cleansing	Infusion		1		
*Nepeta cataria* L.* LE 01079358, LE 01063476	RU *melissa*	S *melissa* (RU)		**2**		**2**
Aerial parts	Sedative	Infusion		1		2
	Sleeplessness			1		
*Origanum vulgare* L. dsPCH19-003, dsPCH19-008, dsPCH19-017	RU *dushitsa, bogoroditskaia travka*	S *dushitsa* (RU)	**3**	**6**		**3**
Aerial parts	Asthma	Infusion	1			
	Bath for babies	Infusion, bath	1			
	Cold	Tea		2		
	Good for health	Infusion	1			1
	Headache			1		
	Heart problems			1		1
	Sedative	Tea		1		
	Sudorific	Infusion		1		
Inflorescences	Sedative					1
*Prunella vulgaris* L.	RU *gorlianka*			**2**		
Aerial parts	Antiseptic for cold	Infusion, rinse		1		
	Throat inflammation	Infusion		1		
*Thymus serpyllum* L.	RU *chabrets*		**2**	**1**		
Aerial parts	Cough	Infusion	1			
	Heart problems		1			
	Rheumatism			1		
Linaceae			**1**		**1**	
*Linum usitatissimum* L.^†^	RU *len*	S *len* (RU)	**1**		**1**	
Stalks	Boils	Fiber rubbed with soap, topical application	1			
	Knee pain	Topical application			1	
Lycopodiaceae			**1**			
*Lycopodium clavatum* L.	RU *likopodii, deriaga*		**1**			
Spores	Wounds	Topical application	1			
Lythraceae			**2**	**1**		
*Punica granatum* L.^‡^	RU *granat*		**2**	**1**		
Peels	Diarrhea (also in children), stomach problems	Infusion	2	1		
Malvaceae			**6**	**10**	**3**	**7**
*Tilia cordata* Mill.* LE 01063409, dsPCH19-022, dsPCH19-032	RU *lipa*	S *lipa,* pähn, pähnapuu	**6**	**10**	**3**	**7**
Flowers	Cold	Infusion	6	4	3	3
	Cold in children			1		1
	Cough			1		1
	General sickness			1		
	Fever			1		
	Heart problems					1
	Sedative					1
	Sudorific	Infusion, tincture		2		
Myrtaceae			**1**			
*Acca sellowiana* (O.Berg) Burret^‡^	RU *feikhoa*		**1**			
Fruit, peels, leaves	Thyroid gland problems	Infusion	1			
Oleaceae			**3**	**4**	**2**	
*Olea europaea* L.^‡^	RU *olivkovoe maslo* (olive oil)			**1**		
Oil	Wounds	Ointment, topical application		1		
*Syringa vulgaris* L.* LE 01063458	RU *siren’*	S *siren’* (RU)	**3**	**3**	**2**	
Flowers	Diarrhea	Infusion			1	
	Diarrhea in children				1	
	Joint problems	Tincture, topical application	1	3		
	Knee pain		1			
Leaves	Diarrhea in children	Infusion	1			
Onagraceae				**2**		**3**
*Epilobium angustifolium* L. dsPCH19-015	RU *ivan-chai, kiprei*	S *ivan-chai* (RU)		**2**		**3**
Aerial parts	Good for the heart	Infusion				1
	Low blood pressure					1
	Sedative	Fermented, infusion				1
Flowers	Heart problems	Infusion		1		
Leaves	Analgesic			1		
Orchidaceae			**1**			
*Orchis militaris* L.	RU *yatryshnik*		**1**			
Root	Male diseases	Tincture	1			
Paeoniaceae				**2**		
*Paeonia officinalis* L.^†^	RU *pion*			**2**		
Petals	Female problems	Infusion		2		
Papaveraceae			**6**	**7**	**3**	**1**
*Chelidonium majus* L.	RU *chistotel*	S *chistotel* (RU)	**6**	**7**	**3**	**1**
Aerial parts	Bath for babies	Decoction, infusion	1		3	
	Organism cleansing	Infusion, tincture		2		
	Skin irritation	Decoction, tincture	1	1		
	Wounds	Tincture		1		
Latex	Warts	Topical application	4	3		1
Pinaceae			**1**	**3**	**1**	**2**
*Picea abies* (L.) H.Karst		S *el’* (RU), kuus’				**1**
Twigs	Leg vein problems	Decoction				1
*Pinus sylvestris* L.	RU *sosna*	S *sosna* (RU), petäi	**1**	**3**	**1**	**1**
Buds	Cough, tuberculosis	Infusion		2		
Shoots	Cold	Decoction, infusion	1		1	
	Immunity	Infusion		1		
Twigs	Leg vein problems	Decoction				1
Piperaceae			**1**	**1**	**1**	
*Piper nigrum* L.^‡^	RU *(chernyi) perets*	S pipar	**1**	**1**	**1**	
Seeds	Diarrhea	Raw	1	1		
	Stomachache	Tincture			1	
Plantaginaceae			**12**	**17**	**11**	**3**
*Plantago major* L. LE 01063457	RU *podorozhnik*	S *podorozhnik* (RU)	**12**	**17**	**11**	**3**
Flower and stem	Sedative	Infusion			1	
	Upset stomach				1	
Leaves	Burns	Topical application			1	
	Cough	Infusion		4		
	Cuts	Topical application	1	2	2	1
	Diarrhea	Infusion		1		
	Leg vein problems	Topical application		1		
	Skin inflammation				1	
	Stomachache	Infusion		1	1	
	Stomach problems			1		
	Upset stomach			1		
	Wounds	Topical application	11	6	4	2
Poaceae			**1**			
*Hordeum vulgare* L.^†^	RU *yachmen’, zhichina*		**1**			
Seeds	Goopy eyes	Infusion	1			
Polygonaceae			**5**	**7**	**2**	
*Polygonum aviculare* L. LE 01063454, LE 01063423	RU *sporysh*		**3**	**2**		
Aerial parts	Female problems	Infusion		1		
	Kidney problems		1	1		
	Menorrhagia		1			
	Stomachache		1			
*Rheum rhabarbarum* L. *	RU *reven’*			**2**		
Stalks	Digestion recovery after oncology treatment	Jam, decoction		2		
*Rumex acetosa* L. * LE 01063414	RU *kislitsa*	S *kislitsa* (RU)	**1**		**1**	
Aerial parts	Diarrhea	Infusion	1		1	
*Rumex confertus* Willd	RU *konskii shchavel’*	S *konskii shchavel’* (RU)	**1**	**2**	**1**	
Aerial parts	Diarrhea	Infusion		1	1	
Leaves			1			
Root, seeds				1		
Primulaceae			**2**		**1**	
*Primula veris* L. dsPCH19-027	RU *pervotsvet, petushki*	S *petushki* (RU)	**2**		**1**	
Flowers	Cough	Infusion			1	
	Good for health	Snack	1			
Root	Cough	Tincture	1			
Ranunculaceae			**1**			
*Anemone nemorosa* L.	RU *vetrenitsa*		**1**			
Aerial parts	Inflammation	Topical application	1			
Rosaceae			**39**	**45**	**20**	**14**
*Agrimonia eupatoria* L.	RU *repeshok*			**1**		
Aerial parts	Good for health	Tincture		1		
*Alchemilla vulgaris* auct. (coll.) LE 01063498	RU *manzhetka*	S *manzhetka* (RU)	**2**	**3**		**1**
Aerial parts	Female problems	Infusion	1	2		
	Good for health			1		
	Headache		1			
	Thyroid gland problems					1
*Aronia melanocarpa* (Michx.) Elliott* LE 01063477	RU *chernoplodka, riabina chernoplodnaia*			**3**		
Fruit	Heart problems	Jam, jam infusion		1		
	High blood pressure			2		
*Comarum palustre* L.	RU *sabel’nik*			**3**		
Aerial parts	Joint problems	Tincture: oral or topical		2		
Root	Joint problems	Tincture: topical		1		
*Crataegus* spp. (including *C. submollis* Sarg. LE 01063511)	RU *boiaryshnik*		**1**	**2**		
Fruit	Coronary heart disease	Tincture	1			
	Heart problems	Infusion, tincture		1		
	Medicine	Infusion		1		
*Filipendula ulmaria* (L.) Maxim	RU *tavolga, labaznik*			**4**		
Aerial parts	Good for heart	Infusion		1		
Flowers	Instead of aspirin			1		
	Kidney stones			1		
	Tonic			1		
*Fragaria vesca* L. LE 01063496	RU *zemlianika*	S *zemlianika* (RU), metsmaasikas	**1**	**2**	**2**	**5**
Flowers	Heart problems	Infusion				1
Fruit	Cough	Tincture				1
	Cough in children					1
	Immunity	Frozen, raw snack		2		
Leaves	Cold	Infusion			1	
	Cough				1	
	Heart pain, heart problems		1			2
*Malus domestica* Borkh.^†^	RU *yabloki*			**2**		
Fruit	Headache	Apple vinegar, topical application		1		
	Tonic	Apple vinegar		1		
*Potentilla argentea* L. dsPCH19-016	RU –			**1**		
Aerial parts	Nerve restoring	Infusion		1		
*Potentilla erecta* (L.) Raeusch. LE 01063425	RU *kalgan, lapchatka, gusinye lapki, uzik*	S *kalgan* (RU)	**11**	**4**	**3**	
Root	Bile expelling	Infusion, tincture	2			
	Female problems	Tincture		1	1	
	Heart problems		1			
	Menorrhagia			1		
	Medicine				2	
	Rheumatism	Decoction				
	Stomach problems	Infusion, tincture	7			
	Tonsillitis, gum problems	Rinse				
	Upset stomach	Tincture	1	2		
*Prunus padus* L.	RU *cheremukha*	S *cheremukha* (RU)	**4**	**2**	**2**	
Flowers	Wounds	Tincture, topical application		1		
Fruit	Diarrhea	Dried, snack, or infusion	4	1	1	
	Sudorific	Snack			1	
*Rosa* sp. dsPCH19-014, dsPCH19-030	RU *shipovnik*	S *shipovnik* (RU)	**1**		**1**	**1**
Fruit	Good for health	Infusion			1	
	Medicine		1			
Root	Kidney stones	Infusion				1
*Rubus chamaemorus* L.	RU *moroshka*		**1**			
Sepals	Male diseases	Infusion	1			
*Rubus idaeus* L. *	RU *malina,* vabarnat (S), varik (S)	S *malina* (RU), vabarnas, varikkas	**18**	**16**	**9**	**5**
Aerial parts with leaves and fruit	Blood dilution	Infusion		1		
	Cold			1	2	
	High blood pressure			1		
	Instead of aspirin			1		
	Sudorific			1		
Fruit	Blood dilution	Jam	2			
	Cold	Frozen, infusion, (raw) jam, jam infusion	9	6	3	2
	Cold prophylactic	Infusion				1
	Diuretic				1	
	Fever	Jam		1		
	Instead of aspirin		1			
	Sudorific		1		1	1
Root	Diarrhea in children	Infusion	1			
Twigs (in winter)	Anti-inflammatory	Infusion		1		
	Cold		3	2	2	1
	Sudorific		1			
	Vitamins in winter			1		
*Sorbus aucuparia* L. LE 01063446	RU *riabina*	S *riabina* (RU)		**4**	**3**	**2**
Fruit	Cholesterol balance	Dried and parboiled snack				1
	Cold	Snack, raw jam, infusion		2		
	Good for health	Infusion			2	
	Headache	Raw jam		1		
	Laxative	Wine, infusion		1		1
	Medicine	Dried			1	
Rutaceae				**2**		
*Citrus limon* (L.) Osbeck^‡^	RU *limon*			**2**		
Fruit	High blood pressure	Raw		1		
Peels	Hangover	Infusion		1		
Salicaceae			**1**	**2**		
*Salix acutifolia* Willd. LE 01063470	RU *verba*		**1**	**1**		
Flowers	Good for health	Eaten on Easter	1			
Twigs	Leg pain	Whip		1		
*Salix caprea* L. LE 01063469	RU *iva*			**1**		
Leaves	Wounds	Topical application		1		
Sapindaceae				**7**		**2**
*Acer platanoides* L. LE 01063411	RU *klion*	S *klion* (RU)		**1**		**1**
Sap	Diuretic	Raw		1		
Twigs	Leg vein problems	Decoction				1
*Aesculus hippocastanum* L.	RU *kashtan*	S *kashtan* (RU)		**6**		**1**
Flowers	Joint problems	Tincture, topical application		1		
	Vein problems	Tincture, topical application		1		
Seeds	Back pain	Put in pocket		**1**		
	Knee pain	Tincture, topical application		1		
	Leg pain	Tincture, topical application		1		
	Sinusitis	Topical application				1
	Vein problems	Tincture, topical application		1		
Saxifragaceae				**1**		
*Bergenia* sp.	RU *badan*			**1**		
Aerial parts	Bruises	Topical application		1		
Solanaceae			**4**	**4**	**3**	**1**
*Capsicum annuum* L.^†^	RU *perets krasnyi*	S *perets* (RU)	**1**		**2**	
Fruit	Cold	Tincture	1		1	
	Diarrhea				1	
*Solanum tuberosum* L.^†^	RU *kartofel’, kartoshka*	S *kartofel’* (RU)*, kartoshka* (RU)	**3**	**4**	**1**	**1**
Shoots	Diabetes	Infusion		1		
	Eye irritation	Infusion or soft tincture, drops		1		
Tubers	Abscessed wound	Raw, topical application	1			
	Burns	Raw, topical application	1			
	Cold	Boiled unpeeled, vapor			1	1
	Cough	Boiled unpeeled, vapor	1			
	Hemorrhoids	Raw, suppository		1		
	Joint problems	Raw, compress		1		
Sphagnaceae				**1**		
*Sphagnum* sp.	RU *mokh*			**1**		
Aerial parts	Wounds	Topical application		1		
Theaceae				**5**		
*Camellia sinensis* (L.) Kuntze^‡^	RU *chai, zelenyi chai*			**5**		
Leaves	Diarrhea	Infusion		1		
	Diarrhea in children	Infusion		1		
	Diuretic	Infusion		1		
	Eye irritation	Infusion, drops		1		
	High blood pressure	Infusion		1		
Urticaceae			**2**		**1**	**2**
*Urtica dioica* L. LE 01063436	RU *krapiva*	S *krapiva* (RU)	**2**		**1**	**2**
Aerial parts	Arm pain	Whip	1			
	Back pain	Dried, within steam bath whisk			1	
	Good for health, organism cleansing	Infusion				2
	Joint problems	Parboiled, steam bath whisk, topical application	1			

No mark = wild, * = wild or cultivated, † = cultivated, ‡ = purchased

### Top 21 taxa

**Fig. 2:**
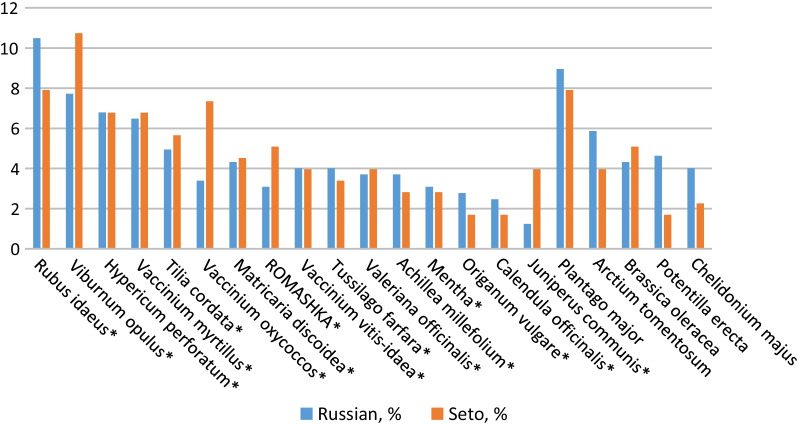
Top 21 taxa reported by Setos and Russians. Percentage (%) of plant uses by ethnic group. (*) = name in Seto was recorded

Figure 2 shows uniformity in the cross-cultural distribution of uses of the top 21 taxa used in Pechorsky District. Among the plants more frequently reported by Setos, *Viburnum opulus* is used for cough and high blood pressure, *Vaccinium oxycoccos* is used for fever, heart problems, headache, and CO poisoning, and *Juniperus communis* is applied as a bath whisk for joint problems. The use of *Potentilla erecta* to alleviate diarrhea and more generally stomach problems was more frequently mentioned by Russians. Only one plant, *Brassica oleracea*, which is used topically to treat headache and joint problems, is cultivated, while the others are sourced in the wild.

Seto names were reported for the majority of the most used plants, except for *Plantago major*, *Arctium tomentosum*, *Brassica oleracea*, *Potentilla erecta*, and *Chelidonium majus*.

### The role of family and social factors in plant acquisition and use

Table 6 presents the average number of plants and plant uses reported by the study participants of various ages. It shows that the largest number of medicinal plants and their uses were reported by people born in the 1940s—the post-war generation who observed wild plants being used by their parents. However, a biology teacher admitted that the local children were more knowledgeable about forest plants than was required by the school program and they showed their expertise during excursions to the forest included in the curriculum (Seto woman, b. 1950). Indeed, the informants who mentioned plant uses by close relatives (usually parents and grandparents) displayed a higher diversity of plants in their own practice. Usually, grandparents served as such mediators because parents were too busy with work in kolkhoz:

*They [parents] didn’t have time once they entered the kolkhoz. It was Estonia there, so I remember kolkhozes [collectivization]. I was born in ‘42. I remember, when I was in 1st grade they herded them—as we then called it*—*into kolkhoz. So… and in the kolkhoz they worked so much that they could not [celebrate] any holiday, [take] any day off. If you don’t show up at work, then… and my parents were not really poor before the kolkhoz, but quite the contrary. We thought they would even de-kulakize us, deport us somewhere. We were ready for that, so all the more reason why they could not miss a day* (Russian woman, b. 1942).

The history of family use and the level of education appear to be culturally specific in the Seto and local Russians. When we look at the cross-cultural distribution, the difference between those with a history of family use and those without it becomes more evident in the Russian group and much less in the Seto group. The correspondence between plant use and education also seems to vary, especially due to the uneven distribution of interviewees across cohorts. In the Russian group, the highest average number of plants was used by college graduates, followed by those with a secondary education. In the Seto group, however, the interviewees with a secondary education reported the highest number of plants, followed by those with a college education. In both groups, those with a vocational education reported the least number of medicinal plants.

**Table 6:** Average number of plants and plant uses depending on age. Data for groups consisting of only one member is italicized

Decade of birth	Average number of medicinal plants/person	Average number of UI/ person	*N*
The number of plants			
< 1930	3.5	3.5	2
1930s	7.4	11	5
1940s	12.9	18.4	14
1950s	9.7	13.4	22
1960s	9.8	11.9	16
≥ 1970	4	4	3
**Total**	9.8	13.2	62

Category	Average number of medicinal plants/person	Average number of UI/ person	*N*
Relevance of family			
Plant uses by (grand)parent or other close relative mentioned	13.4	18.7	29
No uses from other family members	6.6	8.3	33
**Total**	9.8	13.2	62
Setos			
Family use	9.13	12.53	15
No family use	6.82	8.09	11
Russians			
Family use	18	25.21	14
No family use	6.5	8.45	22

Eucation level / Ethnic group	F (plants/UI)	M (plants/UI)	*N*, F	*N*, M
Distribution of the average number of plants by the level of education				
Primary education				
Russians	*3/3*	–	1	–
Setos	–	–	–	–
Secondary education				
Russians	11/15.6	*4/4*	7	1
Setos	*7/9*	*15/22*	1	1
Vocational education				
Russians	10.6/15	6.2/7.8	3	2
Setos	10.9/14.2	6.4/7.4	4	7
College education				
Russians	12.6/18.2	8.4/10.3	9	3
Setos	10.2/13.7	4/5	9	2
Higher education				
Russians	10.7/14.6	6.7/7.8	7	3
Setos	8.6/10.3	–	2	–

### Reading about plants

Some medicinal uses were learned from the literature, but the participants could rarely refer to particular books, unlike the immigrating population who were more aware of the books that influenced them. Obviously, it is not always possible to identify the uses that were driven by recent literature recommendation and then spread via oral transmission. Nevertheless, we asked our interviewees whenever possible to signal the literature that guided some of their plant uses.

Those who could remember the source of the information referred to the *Vestnik ZOZh* newspaper (‘Bulletin of Healthy Lifestyle,’ from Russian: ‘ЗOЖ, здopoвый oбpaз жизни’). Eight of our interviewees mentioned recipes that they learned from this newspaper. Two Russian women (b. 1954 and 1933) referred to *Vestnik ZOZh* describing the use of the leaves of *Arctium tomentosum* for pain in the knees and arthrosis in the feet. Another Russian woman (b. 1960) cited the use of *Pinus sylvestris* shoots for alleviating cough and asthma. A Seto woman (b. 1968) mentioned the roots of *Rosa* sp. for treating kidney stones. Another Russian woman (b. 1941) recommended putting *Dryopteris filix-mas* leaves under the bed sheets to help with sleeplessness. The list of plants linked to published sources is provided in Table 7.

**Table 7:** Plants and uses suggested by *Vestnik ZOZh* and similar publications mentioned during interviews

Plant	Ethnic group	Use
*Allium cepa* L.	RU	Vein problems
*Aloe* sp.	Seto	Runny nose
*Aloe* sp.	RU	Vision, cataracts
*Arctium tomentosum* Mill.	RU	Knee pain, foot pain, headache
*Calanchoe* sp.	Seto	Runny nose
*Calendula officinalis* L.	Seto	Liver problems
*Cichorium intybus* L.	RU	Liver problems
*Dryopteris filix-mas* (L.) Schott	RU	Sleeplessness
*Hypericum perforatum* L.	Seto	Panacea
*Pinus sylvestris* L.	RU	Cough, asthma
*Rosa* sp.	Seto	Kidney stones
*Salix* sp. (Rus. *verba*)	RU	Leg pain
*Taraxacum officinale* (L.) Weber ex F.H.Wigg.	RU	Back pain

Total UI's for genera and taxa are presented in bold

**Fig. 3:**
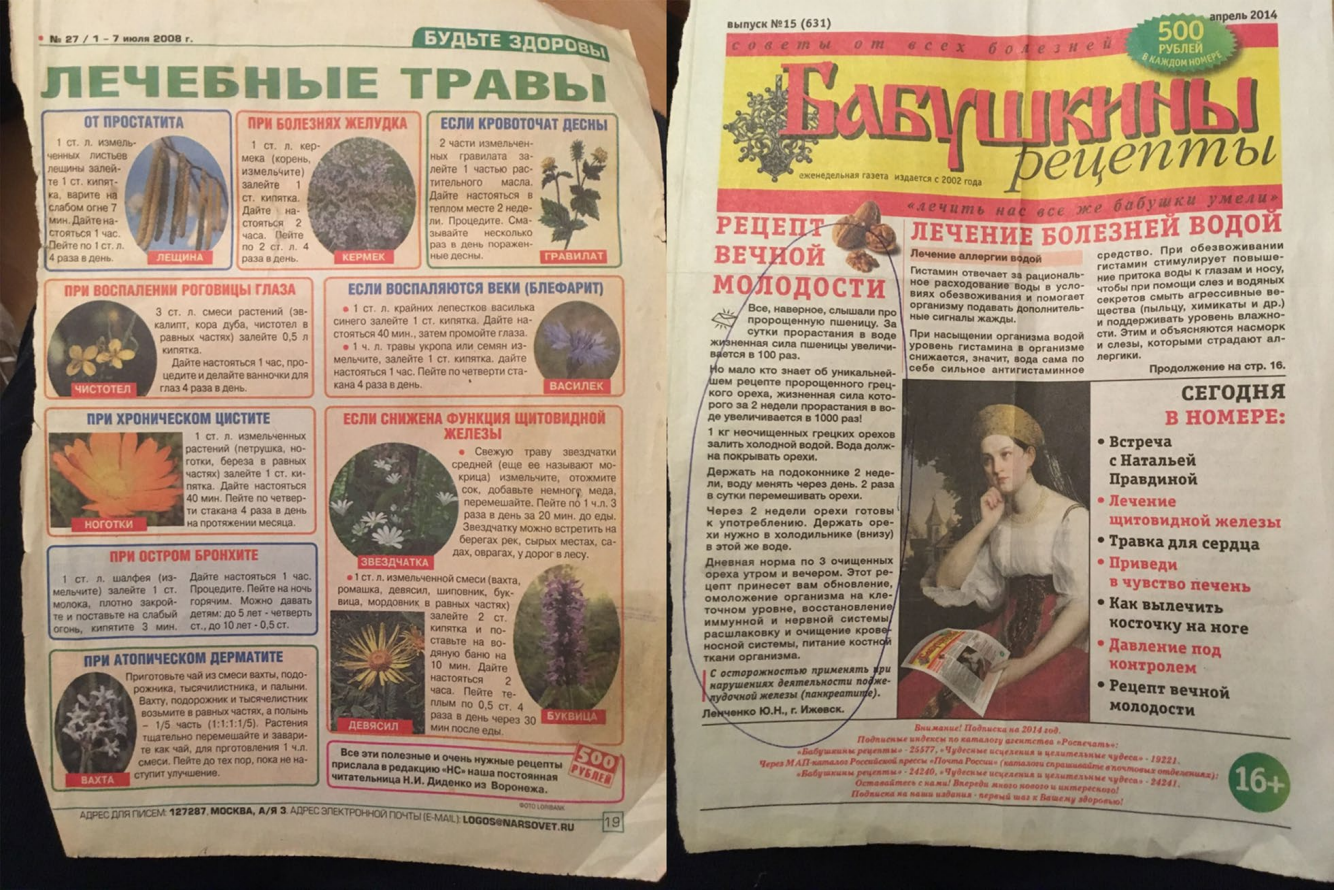
Left: A page from newspaper *Bud’te zdorovy* (Russian ‘Be Healthy’) describing medicinal herbs: *Corylus avellana* L. leaves infusion for prostatitis, *Limonium* sp. root infusion for stomach diseases, *Geum* sp. ointment for gums, poultice from *Chelidonium majus* L., *Quercus robur* L. and *Eucalyptus* sp. infusions, *Centaurea* sp. rinse and *Anethum graveolens* L. infusion for blepharitis, *Calendula officinalis* L., *Betula* sp. and *Petroselinum crispum* (Mill.) Fuss infusion for UTI, *Stellaria media* (L.) Vill. juice and *Inula helenium* L., *Menyanthes trifoliata* L., *Matricaria chamomilla* L., *Echinops* sp. and *Stachys officinalis* (L.) Trevis. infusion for the thyroid gland support, *Salvia officinalis* L. infusion for bronchitis, *Menyanthes trifoliata* L., *Plantago* sp., *Achillea millefolium* L. and *Artemisia* sp. tea for atopic dermatitis. Right: Front page of the newspaper *Babushkiny retsepty* (Russian ‘Granny’s recipes’) with a ‘recipe of perpetual youth’ using germinated walnuts

*Vestnik ZOZh* was founded in 1992 as an appendix to *Sovetskii sport* (Russian ‘Soviet sports’) and became an independent edition in 1998. The newspaper gained a dubious reputation among the general public for mixing the advice of medical doctors for a healthy lifestyle together with letters from subscribers offering anecdotal evidence for the use of home cures that were not supported by scientific research [55, 56]. The ironic perception of these cures can be illustrated in a phrase from the humor column of one of the newspapers shown to us by one of the participants: ‘Folk medicine is when 70-year-olds are treated using the recipes of those who lived for 30 years.’ The main readership of the newspaper is retired people who suffer from chronic diseases and at the same time cannot access qualified medical advice [56]. The name has become the epitome of poor-quality home cures that are used as a last resort for chronic illnesses. Figure 3 demonstrates recipes sent by readers to the various self-care newspapers.

Some interviewees described literature-driven plant uses as potential but unimplemented. For example, a Russian woman born in 1942 discussed with us several recipes that she indicated for personal use and even made the preparations but had not yet tried them, such as *Allium cepa* L. peels boiled in water and used for vein dilatation or joint pain. However, a tincture that was prepared following another recipe and forgotten about for three years was eventually used to a beneficial end: *I read that the flowers of dandelion can be infused in vodka and then [used] for pain in the small of the back. … Then it sat for three years maybe. Then I had liver pain and [when I used the remedy] everything ceased to hurt all at once*.

### Diachrony of disease categories

**Fig. 4:**
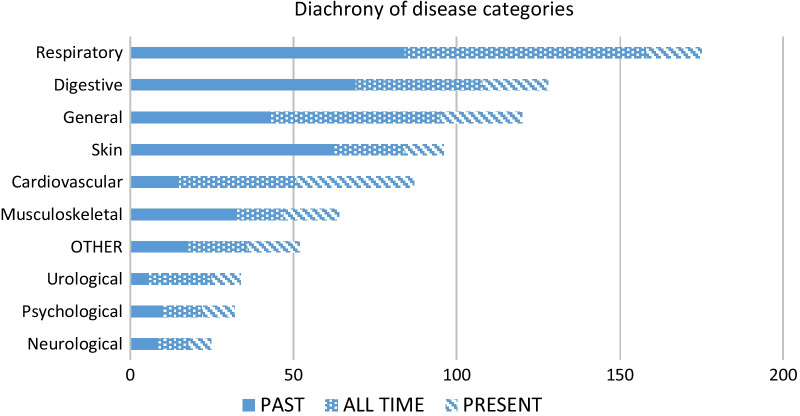
Disease categories distributed over time

The majority of ailments treated by herbs are spread over the respiratory, digestive, and general (fever) disease categories. The category of skin diseases contains the large majority of abandoned uses, which can be explained by improvements in hygiene. In contrast, the cardiovascular category features an important number of newly acquired remedies. Although a significant number of the uses in this category were recorded from only two people (15 and 8 UI), they can nevertheless be a sign of increased vigilance toward these types of diseases. Each category, however, contains a number of recently abandoned uses that is proportional to the total number of uses in their respective categories. In several disease categories, such as digestive, musculoskeletal, general, psychological, and respiratory, interviewees reported uses from long ago, i.e., the generation of their grandparents. The distribution of disease categories over time is presented in Fig. 4.

**Fig. 5:**
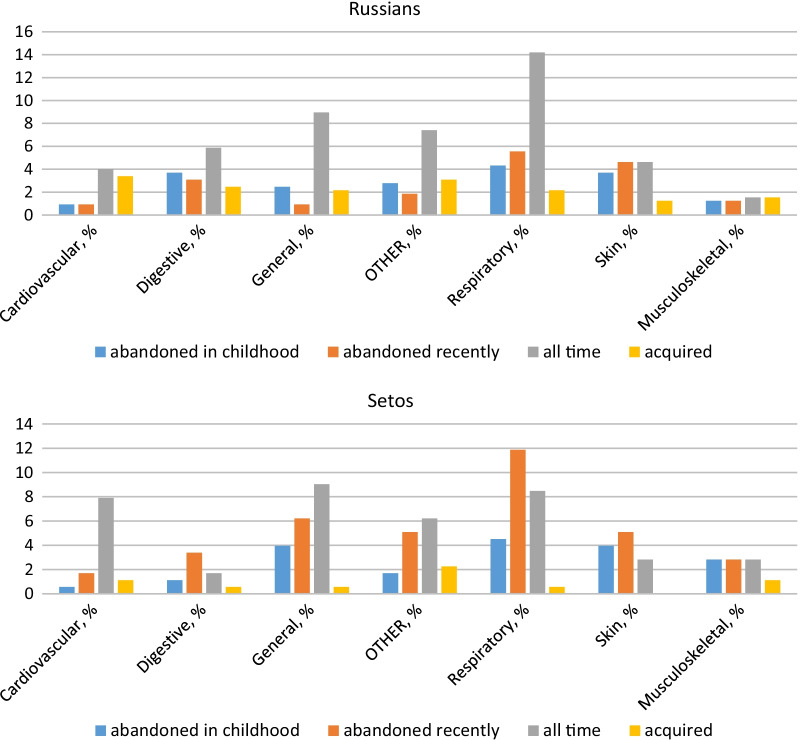
Cross-cultural comparison of the diachronic distribution of disease categories. Percentage of all UIs within an ethnic group. Acquired = adulthood, only now

The diachronic distribution of disease categories in the two ethnic groups is uneven (Fig. 5), demonstrating a developing tradition in the case of Russians and a conservative and eroding one in the case of Setos. In the Russian group, the majority of uses belong to the ‘all time’ use category and each disease category contains a number of newly acquired uses. In the Seto group, all categories demonstrate a number of abandoned uses, while new ones are limited. The majority of permanent uses among Setos belong to the general and cardiovascular disease categories, but most uses in the respiratory and digestive categories were abandoned.

### Cross-border analysis

**Fig. 6:**
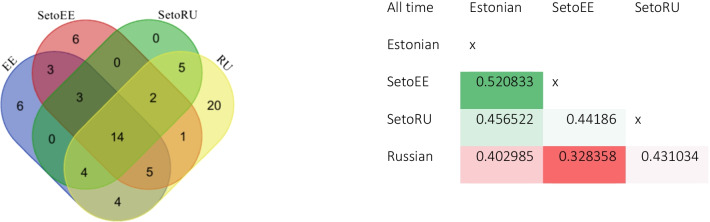
Right: Plant taxa used by Estonians (EE), Estonian Setos (SetoEE), Russian Setos (SetoRU), and Russians (RU). Plants mentioned more than three times in each group are listed. Left: Jaccard Similarity Index

The cross-border comparison reveals more similarity between Estonians and both Seto groups than between Russians and Setos. Indeed, the Jaccard Similarity Index is highest between Estonians and Estonian Setos (0.52) and Estonians and Russian Setos (0.45), while the highest dissimilarity is observed between Russians and Estonian Setos. At the same time, the Seto intraethnic similarity index is quite low (0.44). The Russian group clearly stands out for the number of plants utilized: 55 in the Russian group, 39 in the Estonian group, 34 in the Seto group from Estonia, and only 28 in the Seto group from Russia (Fig. 6).

The analysis of the use of plants by type (wild, cultivated, purchased) revealed a strong preference for wild plants in Russia among both Setos (86%) and Russians (80%) (Table 8). Both studied groups on the Estonian side of the border seem to have a greater predisposition for remedies from cultivated plants (more than 30% in each group), although the proportion of wild medicinal plants is still high (more than 60%).

**Table 8:** Medicinal plants by type

Type	Estonians	Setos (Estonia)	Russians	Setos (Russia)
Purchased	3.2%	3.8%	4.2%	0.4%
Cultivated	35.9%	33.5%	15.7%	13.4%
Wild	60.9%	62.7%	80.0%	86.2%
**All medicinal plants that occur in the garden**	*69.57%*	**74.24%**	**62.38%**	**71.21%**
Non-cultivated (synanthropic) medicinal plants in the garden	27.54%	33.33%	29.70%	37.88%
**Medicinal plants collected in the wild**	**30.43%**	**25.76%**	**37.62%**	**28.79%**

The picture becomes more nuanced when we look at the more detailed plant categories (Table 8). The cross-border division in species selection and uses is evident. Yet, for all groups, from about 30 to 40% of medicinal plants that occur in the garden are wild. In each ethnic group, the proportion of wild plants that occur in the garden is comparable to the plants sourced in other habitats: in particular forests and meadows. The Seto groups in Russia and Estonia reported slightly higher numbers of wild plants that are found in the garden.

The most popular wild plants that occur in the garden and were frequently reported by at least three groups included: *Plantago major* L., *Betula* sp., *Achillea millefolium* L., *Matricaria discoidea* DC., and *Hypericum* sp. Both Estonian groups frequently mentioned *Picea abies* (L.) H.Karst. Both groups in Russia also reported *Tussilago farfara* L., *Arctium tomentosum* Mill., and *Chelidonium majus* L. The wild plants that occur in cultivations were *Tilia cordata* Mill. (in all four ethnic groups), *Mentha* sp., *Ribes nigrum* L., *Rubus idaeus* L., and *Viburnum opulus* L. (the last two mostly in Russia). The cultivated plants that grow wild (garden-CW) and were used in at least three groups were *Rosa* sp., *Nepeta cataria* L., *Syringa vulgaris* L., and *Aronia melanocarpa* (Michx.) Elliott. The Russian group also frequently mentioned *Leonurus quinquelobatus* Gilib. which, according to Efimov and Konechnaia [30], was dispersed throughout the Pskov region after large scale cultivation. The most common cultivated plants were *Brassica oleracea* L., *Allium cepa* L. (mostly in Estonia), *Solanum tuberosum* L., and *Calendula officinalis* L.

**Fig. 7:**
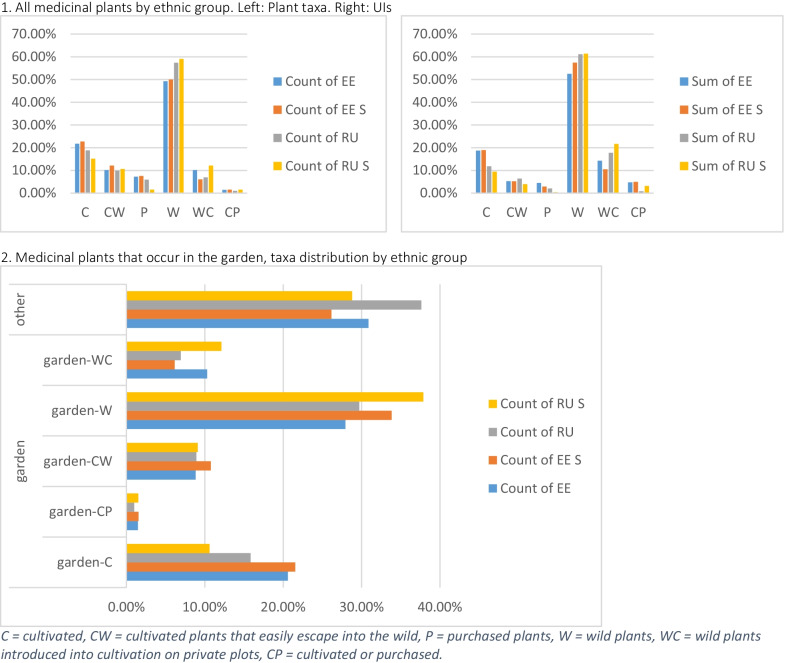
Medicinal plants that occur in the garden and in the wild, % by ethnic group

Both Seto groups rely more than their immediate neighbors on the wild plants that grow next to their houses, and at the same time use fewer medicinal plants from the wild, indicated as ‘other’ (Fig. 7, bottom). The four species from this category that overlap among Setos are also frequently used by Estonians and Russians: *Plantago major* L., *Hypericum* sp., *Matricaria discoidea* DC., and *Arctium tomentosum* Mill.

### Diachronic comparison

**Fig. 8:**
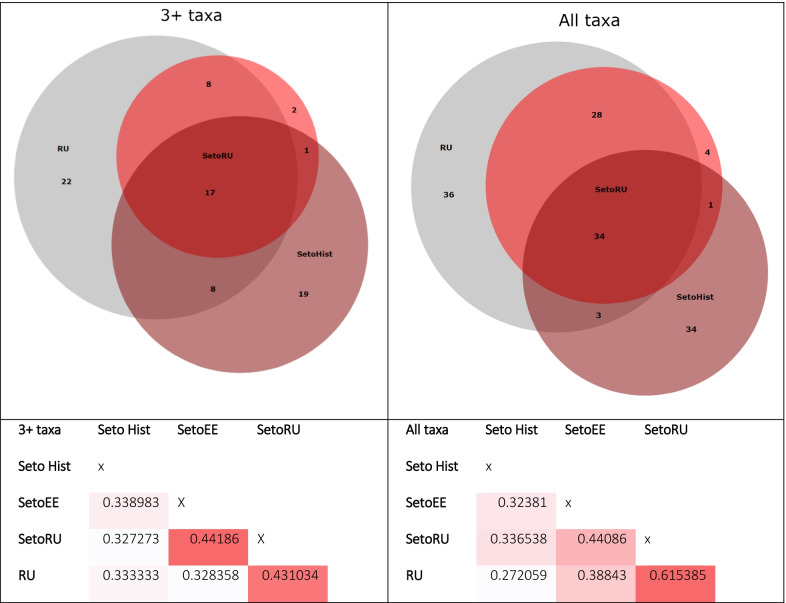
Plant list comparison: Historical Seto uses obtained in [45] = Seto Hist, Seto and Russian uses—field data. 3 + taxa = plants mentioned more than three times in each group are included in the analysis

When compared to the historical data collected in the region in the 1930s, both Russian and Estonian Setos appear to be quite disparate: Jaccard Similarity Indexes range from 0.34 for Estonian Setos to 0.32 for Russian Setos in the 3 + taxa comparison and remain at roughly the same level for both groups in the comparison of all taxa (Fig. 8). The Russian group demonstrates similar level of dissimilarity, being 0.33 in the 3 + taxa comparison and 0.27 in the all taxa comparison.

The level of Seto interethnic similarity is low, varying from 0.37 to 0.43. The highest similarity is observed between historical Seto uses and those of the Russian group—0.41 for the 3 + taxa and increasing up to 0.61 for the all taxa comparisons. This could mean that although the set of key plants is preserved in Russian Setos in the same volume as in Setos across the border, they borrow the new singular plant uses from the neighboring local Russians.

## Discussion

### Seto medicine: stones, springs, and ether

The data on traditional Seto medicine is scarce. The materials gathered during the ethnographic expedition of Leonid Zurov and Boris Vilde in 1937–1938 provide information about spiritual landscape objects such as stones, springs, and trees that were involved, among other things, in magical practices aimed at improving health [41, 57]. For example, Zurov mentions a stone that was used by Seto women to treat infertility (Ibid.). Others were used to help with leg pain and vertebral column problems, as it was believed that St. John or St. Peter had sat on them [41]. The stones were usually associated with the appearance of Christian saints. In Russian tradition, water from the depressions in these stones, so-called god’s footprints, was considered to be able to heal diseases like arthritis, wounds, or sprains [58, 59].

**Fig. 9:**
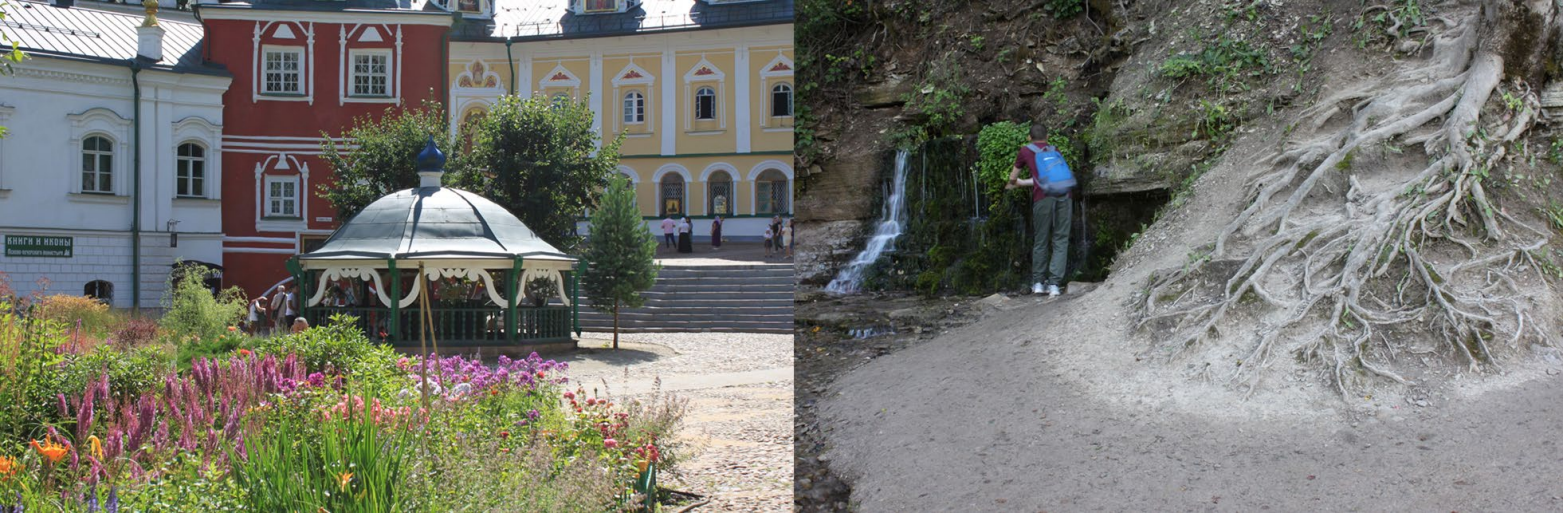
Left: Holy spring in a chapel in the yard of Pskov-Pechory Monastery. Right: Slovenskiye springs in Izborsk, Pechory District

Springs and berry patches were the domain of female ecological knowledge [57]. The two main spiritual centers of Pechorsky District, Pskov-Pechory Monastery (Fig. 9) and Maly churchyard, were built on the springs whose waters are believed to have beneficial properties. Our field data contain references to ‘eye springs’ made by a Russian woman (b. 1933) and a Seto woman (b. 1960, see in a quote below).

*For the eyes… for the eyes, they kept telling us when we were kids that there is an eye spring. ‘Go wash your eyes’* (Seto woman, b. 1960).

Another Russian woman remembered using the water from such a spring herself:

*If I tell you, you will laugh. I went to that Nikander poustyn [70 km from Pskov], still going there, there is an eye well. … There are many wells, and this one is for the eyes. A Kazan Mother of God icon is hanging there. … I went there on foot [about 7 km], over the swamp… And then I took this water. It really [helped]. Or maybe I was still young enough so that it helped me. So I washed my eyes then and could read without glasses* (Russian woman, b. 1945).

The public availability of ether made it the most popular folk remedy among Setos, for which they were notoriously known even by the first ethnographers [42]. Two of our Seto informants, a man born in 1971 and a woman born in 1965, remembered using ether in treating high fever in their childhood.

### Patterns of plant use

The comparison of the top families and taxa used revealed interesting details, even though the overall set of taxa seems to be uniform across Russians and Setos.

The top families are distributed evenly in the Seto group, while in the Russian group Asteraceae (107 UI, 17 taxa) far outnumbers Rosaceae, the second most common family (84 UI). The same preference for the Asteraceae family in the medicinal domain has been recorded for other Slavic communities: those living along the Ukraine–Poland border in the nineteenth century [60], modern day Hutzuls [61], and Slovenes historically residing in Italy [62]. Indeed, Asteraceae contains the largest number of taxa that are included in the ethnobotanies of various cultures. At the same time, Asteraceae is the most numerous family of northwestern Russia, containing 392 species (including 95 species of *Taraxacum* genus) and being much larger than second-largest Poaceae with 207 species [63].

We compared our field data with the records made by Lebedeva [64, 65] who worked in the region in several previous years. Although a different methodology was applied during her fieldwork, we could nevertheless compare the lists of plants. She described 15 taxa that coincide with our data: *Alnus incana* (L.) Moench, *Arctium minus* (Hill) Bernh. (in our data *Arctium tomentosum* L.), *Artemisia* sp., *Betula* sp., *Equisetum* sp., *Juniperus communis* L*.*, *Lepidotheca suaveolens* (= *Matricaria matricarioides*, most likely the same ethnotaxon as *Matricaria discoidea* DC in our data), *Mentha arvensis* L., *Oxycoccus* spp., *Pinus sylvestris* L., *Plantago major* L., *Plantago media* L., *Quercus robur* L., *Rubus idaeus* L., *Vaccinium myrtillus* L., and *Vaccinium vitis-idaea* L. (fruit and leaves). The medical applications also coincided (e.g., *J. communis* bath whisk for radiculitis, *P. sylvestris* shoots with honey for tonsillitis), although Lebedeva provides some interesting details; for example, the leaves of *Plantago* spp. should be rubbed with the one-day cream (that accumulates on the top of milk during 24 h) before topical application.

*Viburnum opulus*, *Vaccinium oxycoccos*, and *Juniperus communis* are the only species that are more intensively used in the Seto group than in the Russian group. While *Juniperus communis* is traditionally used in the region, *Viburnum opulus* seems to be borrowed from Russians. The presence of *Vaccinium oxycoccos* is interesting because unlike the other two species it was absent from the list of medicinal plants of Estonia of the nineteenth century [66], and it seems that the collection (and medical use) of this berry was popularized in Estonia during Soviet times [67]. However, one use of *V. oxycoccos* had already been recorded by Vilbaste in 1930. Thus, the Seto population might have acquired this plant by that time. Historically in Russia, cranberry juice with honey was used to alleviate fever and crushed cranberries were used to treat impetigo [68].

The use of *Viburnum opulus* was the most significant in the Russian Seto group (19 UI, 7.5% of all Seto uses), while being absent from the data for Estonian Setos. It was also present in the Russian (15 UI, 4%) and Estonian (6 UI, 1.6%) groups. In the 1950s, a medicinal use of *Viburnum* fruits was recorded [69]: Steamed with honey or in the form of juice they were used in the treatment of whooping cough. The juice was applied topically to treat scrofula in children. The use of *Viburnum* against cough and skin problems was recommended in the local newspaper [70].

The most salient difference in the Russian group is *Potentilla erecta*, which has been present in traditional Russian medicine since the nineteenth century and was actively popularized in Soviet wild medicinal flora guides. In Estonia, the use of this plant was recorded in Setomaa (Värska) as early as the 1930s [45], but in our field materials it was only recorded among Estonians and not among Estonian Setos. The Russian field data uses focused around digestive disorders and gynecological bleeding (15 UI), whereas the four Estonian UI mention skin bleeding, inducing childbirth, and toenail fungus. *Potentilla* is known in Estonia by local names (*tedremaran*) as well as by its Russian name (*kalgan*). At the end of the nineteenth century, *Potentilla* tincture was considered by Russians to be powerful remedy for cholera [71].

### Diachrony of disease categories: cardiovascular diseases

The landscape of diseases, that is, diseases recognized on the emic level and cured by local herbal medicine, has changed over time, partly due to improvements in hygiene and the more widespread availability of medical aid. Indeed, the proportion of skin diseases has decreased and the fever ailment in the general category is mostly linked to respiratory diseases. While respiratory and digestive cures are the most frequently applied, there are some new ailments, such as cardiovascular diseases, that reflect the health concerns of the local population.

Cardiovascular diseases are the main cause of death (61%) in Pskov Oblast, which is in line with the global trend, although the proportion and absolute numbers are quite high. As of 2018, the coverage of outpatient clinics by cardiologists in Pskov Oblast was only 25% [72]. The fact that the majority of the rural population live in isolated farmsteads contributes to the reduced accessibility of emergency medical care. Only 77% of patients with acute myocardial infraction and just 30% of patients with ischemic stroke were hospitalized within the therapeutic window (Ibid.). In 2019, a regional program was launched aiming to decrease mortality from circulatory diseases from 1050 to 825 per 100 thousand, at a minimum, by 2024 (Ibid.), while across Russia this number does not exceed 585 per 100 thousand [73].

### Family as an agent of knowledge transmission and the role of formal education

Early plant knowledge acquisition and vertical knowledge transmission play key roles in ethnobotanical knowledge resilience [74, 75]. While traditional gender and family roles can pose certain limitations on access to plant knowledge, learning by doing is the most important part of knowledge acquisition and, just like with language learning, interruptions and delays at this stage can be detrimental to a person’s relationship with plants in the future. However, the precise list of learned medicinal plants can vary and is susceptible to change, adapting to a person’s constitution, habits, accessible habitats, and set of health problems.

As the majority of ethnobotanical studies are carried out in communities with limited education, their observations only discuss the presence or absence of one. They report, more or less unanimously, that education is a negative factor in the preservation of LEK [15]. However, there is an opinion that the contents of education might play a role. Other studies oppose the established point of view claiming that the volume of LEK does not depend on education [18]. Also, it can play a role in knowledge hybridization when patients with a higher education seek remedies in both the pharmaceutical and ethnobotanical domains [76]. Our study was carried out in a highly literate community, where the great majority of inhabitants have at least a secondary education [77]. According to our results, the highest number of plants was used by participants with a secondary (~ 11 UI) or college education: 15.6 UI in the Russian group and 9.8 in the Seto group (Table 6). In contrast, despite the frequently reported use of wild flora guides, people with a higher education provided 6 to 9 UI on average. The most obvious cross-cultural difference is measured at the level of college and higher education where the Russian group demonstrated the use of a wider variety of plants.

The greatest number of plant uses was recorded among people whose occupations included librarian, teacher, local historian, agronomist, and accountant, of which only one, an agronomist, used to be professionally involved with plants but later also worked as a school director. It is tempting to conclude that the largest number of plants was reported by members of the local intelligentsia whose outlook was guided by publications on useful plants that flooded libraries starting in the 1970s. Indeed, the myth of the Soviet reader and the most active reading nation was created in the 1930s, only to be reinforced after WWII [78]. Yet, the importance of books and reading was unquestionable, books were readily accessible and the library network was extensive. Until 1974, for those who worked in a kolkhoz, reading and receiving an education was the only way to gain admission to a college or university and to obtain a passport [79, 80]. Lovell notes that the most widely published magazines of that time (for example, *Nauka i zhizn’,* Russian ‘Science and life’) were devoted to science popularization. They not only described the latest advancements in technological progress but also advised readers on various practical household issues. Similarly, publications on plants varied from direct calls to action for contributors to collect medicinal herbs to more subdued lists of medicinal plant properties, appearances, and habitats. Being the co-creators of the reading nation myth, teachers and librarians were the most aware of popularizing books, including those on practical botany. Childhood exposure to plants, inevitable in a village or small town, combined with access to useful plants guides proved to be especially beneficial to the local intelligentsia.

In all likelihood, the lack of the second component, namely the effective influx of one-to-many book knowledge, rendered the numbers in the Seto group more uniform and, at the same time, the set of plants more stable. Vertical transmission provided a more unified repertoire of plants, more similarity among various levels of education, as well as more uniformity among those who referred to a family model of plant use and those who did not. Moreover, the access of Setos to literature in Estonian was limited. While educational instruction was conducted in Estonian using Estonian textbooks, local libraries and bookshops did not offer any literature in Estonian. Nevertheless, several families mentioned subscribing to Estonian magazines for women, while the border still was only administrative, which provided more information on knitting patterns than on self-medication. One of our interviewees, however, referred to Estonian radio for a medicinal remedy using *Fragaria vesca* L. After 1991, subscribing to Estonian newspapers and magazines was no longer an option. Currently, the Seto community in Russia seems to be dissimilated: Seto presence in each village does not number more than 2–3 people who only have the opportunity to meet during celebrations several times a year, such as the Dormition of the Mother of God on 28 August or Maly (Estonian Mõla) Sunday in July. Seto families keep in contact with their relatives in Estonia, sometimes more than with their physical neighbors in the next village. None of our Seto interviewees discussed medicinal plants acquired from the current practice in Estonia, neither from relatives nor from other sources of information.

## Preference for synanthropic plants across the border

The balance between wild and cultivated medicinal plants in a given community can also indicate important tendencies on various levels. Although cultivation seems to be the most logical choice, it is not always possible due to the ecological properties of certain plants [81]. On the other hand, wild plant harvesting itself can be valued as an identity-building or recreational practice with potential health benefits rooted in maintained contact with nature as well as sourcing useful wild plants [82–84]. While some traditions, over time, tend to choose more easily accessible cultivated plants over the wild ones [66], as in the example of Estonia, other traditions, like that in Belarus, retain a preference for wild flora [85]. Recent studies have shown that the traditional medicine of Russia might prefer wild plants as well [20, 86]. However, what is important for our analysis is not the distinction between wild and cultivated plants, but more the ability of the chosen plants to cohabitate with humans. Our cross-border comparison revealed a stronger preference for wild plants among Russians and Setos residing in Russia compared to Estonians and Setos residing in Estonia. Our finding corresponds to the historical observations made in Estonia indicating that synanthropic plants started to be used more frequently for medicinal purposes at the end of nineteenth century [66]. Field data from neighboring Belarus, however, stress the importance of wild plants over cultivated ones [85]. Globally, the proportion of traditionally used cultivated medicinal plants is reported to be around 20% [87–89]. According to our data, Estonia is above (33–35%) and Russia is below (13–15%) this figure.

Although wild plants constitute the majority of medicinal uses in all studied cultures, almost all of them use the shortcut of choosing the readily available wild plants that occur next to their house, in the garden, or by the side of the road. Indeed, the illnesses that appear as we get older are the pretext for turning to medicinal plants, but the same illnesses may prevent someone from accessing remote habitats. Nevertheless, Setos in both Estonia and Russia spoke more frequently about the easily accessible wild plants growing next to their houses.

Several considerations might help delineate the importance of wild medicinal plants. First, this preference can be linked to their presence in published sources: see, for example, Table 7, listing the plants cited by the interviewees from various publications. Moreover, the publications emphasized the importance of conveniently available anthropophytes, while claiming that it is wild plants whose medicinal properties are more expressed [90]. Although it was not stressed in the first popular Soviet publications on medicinal plants [91], the immediate availability of medicinal plants in the form of weeds became more important later [92], having developed into a separate subcategory in contemporary Russian guides to wild plants [93, 94] and many others.

On the other hand, immediate access of rural inhabitants to the forest, as well as plant literacy preserved by family transmission, enables increased wild plant use. Those who do not have such access, however, can profit from the wild plants sold in pharmacies (at a much lower price than pharmaceuticals), at the local market, or supplied by their network of contacts. Take, for example, the evidence from our Seto interviewee (born 1960) who does not collect herbs but who was able to identify and collect *Hypericum* for her relative. Despite claiming that she does not generally collect herbs, she was able to correctly identify *Hypericum perforatum* L. thanks to its properties that she learned as a child: The red pigment produced by its flowers was used to paint nails by young girls during her childhood:

*I do not collect herbs. Well, only one year I collected zveroboi [St. John’s wort]. I read somewhere, [that you need] to macerate it in olive oil and put it on joints. So, I did and gave it to the mother of my daughter-in-law, she has knee pain* (Seto woman, b. 1960).

## Conclusion

The case study demonstrated cross-cultural as well as cross-border differences among the four studied groups: Setos residing in Russia and in Estonia, and Russians and Estonians living in Russia and Estonia, respectively. By the number of used plants, Russian Setos are similar to Estonian Setos and Estonians, while the set of plants is similar among all four groups. Russian Setos and Russians exhibited a preference for wild plants over cultivated and purchased plants, which is inspired by the overall plant literacy, access to nature, as well as one-to-many knowledge transfer favoring wild plants.

Our data revealed that nature mediators play an important role in LEK transmission and retention. In our case, grandparents rather than parents played the role of mediators, which has helped to maintain the link between generations. The role of education is not that straightforward for LEK. In our sample, apart from the children of herbophilic families, the rural intelligentsia including librarians and teachers reported the highest number of medicinal plants, representing knowledge learned not only from published sources but also from the family. It seems that the academic formalization of knowledge helps to secure the disembodied knowledge obtained in a family.

For Russians, herbal self-medication is an important cultural trait that was partly inspired by the preexisting traditional plant use and partly by the limited access to official medicine and pharmaceuticals caused by the turbulent history of the region. For both groups, Setos and Russians, reading about plants and formalization of the ecological knowledge supported the initial interest sparkled in the family. In the case of Setos, however, the absence of books and print media in Estonia prevented their LEK from evolving. Setos of Pechorsky district reported a more narrow and homogenous set of medicinal plants than that of Setos in Estonia. However, due to fragmentation of the Seto community in Russia and the erosion of horizontal links, their medicinal practice has started to diverge on the individual level and thus be homogenized with the Russian one (see Fig. 8).

We encourage studies focusing on the transformation of local herbal practices in relation to various ethnic and geographic contexts to document current practices and to estimate the persistence of traditional uses and their transformation over time in the context of differing habitats. We also encourage further studies regarding different forms and levels of education in relation to LEK in literate societies to identify successful strategies.

## Data Availability

The datasets used and/or analyzed during the current study will be made available after the project ends (2023).

## Abbreviations

C: Cultivated plants
CO: Carbon monoxide
COVID-19: Coronavirus disease 2019
CP: Cultivated or purchased plants
CW: Cultivated plants that easily escape into the wild
EE: Estonian
F: Female
LEK: Local ecological knowledge
M: Male
N: Number of participants
P: Purchased plants
RU: Russian
S: Seto
SSR: Soviet Socialist Republic
UI: Use instance
UTI: Urinary tract infection
W: Wild plants
WC: Wild plants introduced into cultivation on private plots
WHO: World Health Organization
WWII: Second World War
